# Modeling human embryo adhesion using a microfluidic platform

**DOI:** 10.1126/sciadv.adz2249

**Published:** 2026-07-10

**Authors:** Sofía Zaragozano, María Pardo-Figuerez, Ana Monteagudo-Sanchez, Anna Quirant, Javier Moncayo-Arlandi, Javier Gonzalez-Fernandez, Jaime Llera-Oyola, Petr Volkov, Sara Maggi, Luis Quintero, Francisco Raga, Pablo Grases, Xavier Santamaria, Inmaculada Moreno, Nicolas Plachta, Carlos Simon, Felipe Vilella

**Affiliations:** ^1^ Carlos Simon Foundation, Valencia, Spain.; ^2^ INCLIVA Biomedical Research Institute, Valencia, Spain.; ^3^ University of Valencia, Valencia, Spain.; ^4^ Doctoral Program in Biotechnology, Universitat Politècnica de València, Valencia, Spain.; ^5^ Next Fertility, Valencia, Spain.; ^6^ Department of Pediatrics, Obstetrics and Gynecology, School of Medicine, University of Valencia, Valencia, Spain.; ^7^ Department Ginecología y Reproducción Clínica El Pilar, Barcelona, Spain.; ^8^ Department Ob/Gyn Vall d’Hebron Institut de Recerca, Barcelona, Spain.; ^9^ Department of Cell and Developmental Biology, Institute for Regenerative Medicine, Perelman School of Medicine, University of Pennsylvania, Philadelphia, PA, USA.; ^10^ Department of Obstetrics and Gynecology, Beth Israel Deaconess Medical Center, Harvard Medical School, Boston, MA, USA.

## Abstract

Embryo adhesion represents a critical step of implantation, yet understanding this process has been hindered by the lack of human in vitro platforms that replicate endometrial physiology. Here, we present a dual-channel microfluidic platform containing organoid-derived endometrial epithelium and primary stromal cells. Our model recapitulates important endometrial hallmarks including epithelial polarization, stromal decidualization, extracellular vesicle release, and hormone-induced receptivity. We validated the model using mouse embryos and human blastocysts, where we showed that embryos displayed features of initial adhesion. These included establishment of embryo-epithelial contacts initiated via the polar trophectoderm, inner cell mass repositioning, and lineage reorganization. Moreover, human embryos secreted βhCG indicating a functional trophoblast. Thus, this work provides a platform to study key features of embryo adhesion and endometrial receptivity and disorders affecting embryo-endometrium interactions.

## Teaser

Endometrium-on-a-chip shows detailed human embryo adhesion dynamics.

## INTRODUCTION

The endometrium is the hormonally regulated inner lining of the uterus that undergoes cycling remodeling to become receptive for embryo implantation (*1*). As the earliest event in pregnancy, implantation involves blastocyst apposition, adhesion to the endometrial epithelium, and invasion into the decidua (*1*). Adhesion serves as a limiting step of the periconceptional period influencing long-term health outcomes for both mother and child (*1*, *2*). Insights into human embryo adhesion derive primarily from in vivo studies compiled into the Carnegie stages of human development, a foundational reference in human embryology (*3*). Complementary studies in murine models have elucidated mechanisms underlying embryo structural remodeling during the blastocyst-to-postimplantation transition. These include rosette formation, cavity formation, maintenance of pluripotency, and trophectoderm (TE) polarity loss and cell migration (*4*–*6*). However, important physiological differences between mouse and human implantation, including the site of implantation (abembryonic in mouse versus embryonic in human), the implantation pattern (eccentric in mouse versus interstitial in human), or trophoblast differentiation (giant cells in mouse versus villous cytotrophoblast in human) (*7*) may affect the direct applicability of these findings.

Initial in vitro models using human endometrial epithelial cells (EECs) (*8*) or immortalized EEC lines (*9*) have evolved to the use of three-dimensional (3D) systems (*10*). The development of endometrial epithelial organoids (EEOs) has recently offered the ability to replicate 3D architecture and hormone response (*11*, *12*). Although insightful, these models lack endometrial stromal cells (ESCs), essential for the cross-talk between the embryo and endometrium in the in vivo environment (*11*–*14*). To address this, 3D systems have incorporated EEOs within 3D matrices that support coculture with stromal cells and other cell types (*15*–*17*). However, the architectural constraints of these systems limit the precise observation and characterization of the sequential stages of embryo implantation.

Microfluidic devices enable precise control of cellular interactions, chemical gradients, and mechanical forces, key parameters for recapitulating in vivo physiology (*18*, *19*). They have been successfully used to model hormone responses (*20*), microbiome-host interactions (*21*, *22*), and implantation dynamics within the female reproductive tract (*23*). Major regulatory agencies, including the Food and Drug Administration, Environmental Protection Agency, and National Institutes of Health, increasingly support systems such as organ-on-a-chip models as human-relevant alternatives to animal testing (*24*). Because animal models and static 2D cultures challenge the study of tissue polarity, multicellular communication, and functional secretory activity, these platforms offer superior translational relevance (*18*, *19*). Building on these advances, we developed an endometrium-on-a-chip model using a commercially available microfluidic platform to investigate adhesion using both mouse and human embryos. Our model recapitulates the essential features of the endometrial epithelium, as well as epithelial-stromal interactions. This platform has allowed us to study the events underlying embryo adhesion in mouse and human blastocysts.

## RESULTS

### Development and characterization of an in vitro adhesion dynamics–on–a–chip model

**Fig. 1.**
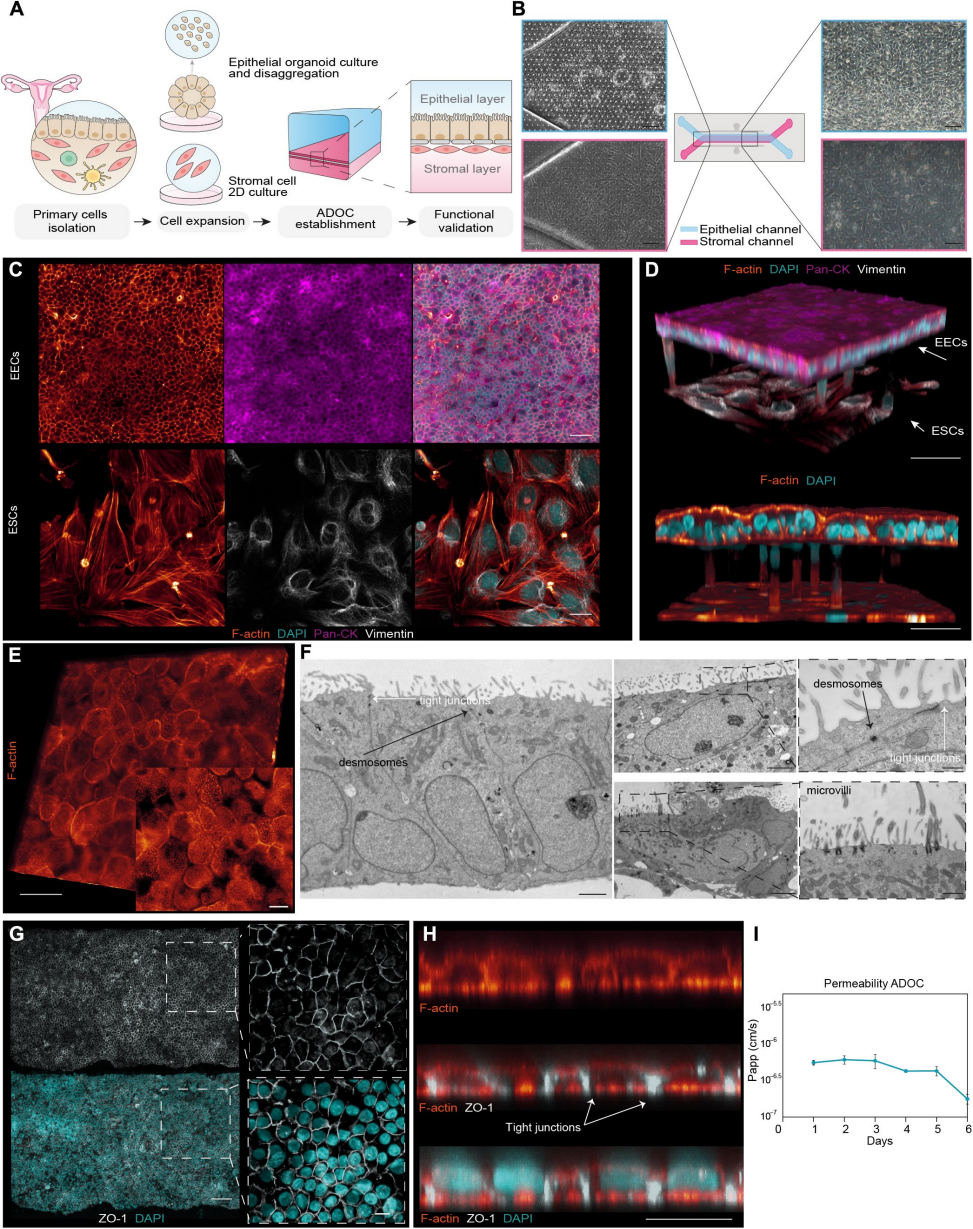
Development and characterization of an in vitro ADOC. (**A**) Schematic overview of the establishment of the endometrium-on-a-chip model. (**B**) Diagram and bright-field images showing the epithelial (blue) and stromal (pink) channels after 6 days of growth. Scale bars, 100 μm. (**C**) Immunostaining of epithelial cells (top) with F-actin (orange), and pan-cytokeratin (purple) and DAPI (blue); scale bar, 20 μm; and stromal cells (bottom) with F-actin (orange), vimentin (white), and DAPI (blue); scale bar, 15 μm. (**D**) Top: 3D blend rendering projection showing the epithelial monolayer (above) and the stromal layer (below) stained with F-actin (orange), panCK (purple), vimentin (white), and DAPI (blue). Scale bar, 30 μm. Bottom: 3D blend rendering showing the transversal cross section of ADOC highlights the columnar morphology of the epithelium and migration of stromal cells toward epithelial cells. Scale bar, 40 μm. (**E**) Epithelial compartment stained with F-actin revealed microvilli as distinct dots. Scale bars, 100 μm. (**F**) Electron microscopy shows desmosomes (black arrows) and tight junctions (white arrows), confirming epithelial cell polarization. Scale bars, 2 μm and 500 nm. (**G**) Immunostaining of tight junctions (ZO1-gray) demonstrates a well-formed epithelial barrier. Scale bar, 100 μm. (**H**) Cross section of the epithelial layer highlights the tight junction (ZO1-gray) deposition within individual cells. Scale bars, 100 μm. (**I**) Permeability assay using cascade blue shows minimal molecular exchange after cell growth in the model (data are presented as the mean ± SD).

To investigate the mechanisms underlying embryo adhesion in vitro, we first developed an endometrium-on-a-chip model by using a commercial microfluidic system (Emulate Inc., Boston, USA) consisting of two channels connected by a 7-μm-pore membrane, allowing communication between upper and lower compartments. We named this model ADOC (Adhesion-Dynamics-On-aChip). ADOC design is based on an epithelial compartment in the upper channel of the system and a stromal compartment in the lower channel (Fig. 1, A and B). To establish the epithelial compartment, organoid-derived EECs were grown as a monolayer in the top channel, while stromal cells were seeded from primary 2D cultures (see Materials and Methods). Following 6 days of culture, both channels showed confluence (Fig. 1B and fig. S1, A to C). Immunofluorescence analysis confirmed that EECs expressed keratins, a marker of epithelial identity (*25*), while ESCs exhibited the expression of the mesenchymal marker vimentin (Fig. 1C). F-actin staining revealed the epithelial monolayer distribution and highlighted the characteristic fibroblast-like morphology of stromal cells (Fig. 1C), which exhibited interdonor morphological variability (fig. S1D). Visualization of the chip organization in 3D showed both epithelial and stromal layers separated by the porous membrane (~50 μm thickness), which can be visualized as the separation between both layers (fig. S1E). EECs grew on the upper face of the membrane and were arranged in a columnar structure (Fig. 1D), recapitulating the native organization of the luminal epithelium (fig. S1F). Quantification of epithelial cell height revealed comparable values between in vivo tissue and ADOC (fig. S1G), confirming that the model preserves the cuboidal-to-columnar morphology characteristic of an epithelial endometrium (*26*). Stromal cells were cultured on the opposite face of the same porous membrane, forming a stromal cell monolayer within the lower channel. As observed, these stromal cells are physically separated from the epithelium but remain functionally coupled through the membrane pores, which permit molecular diffusion and paracrine signaling. Stromal extensions frequently oriented toward the epithelial layer appearing as pillars across the 7-μm pores of the chip membrane and suggesting interaction across epithelial-stromal compartments (Fig. 1D).

Characterization of the epithelial compartment showed apical microvilli in the EEC monolayer, visualized by F-actin (Fig. 1E). Transmission electron microscopy (TEM) confirmed epithelial cell polarization, revealing basally located nuclei surrounded by organelles, and an apical surface densely covered with microvilli (Fig. 1F). We further identified tight junctions and desmosomes (Fig. 1F), crucial for epithelial polarity (*27*). Immunofluorescence for the tight junction protein ZO-1 revealed that cell membranes were in tight contact forming a continuous barrier structure (Fig. 1, G and H), essential for preserving epithelial integrity (*28*). To evaluate the stability and integrity of the epithelial barrier, we measured the apparent permeability (*P*_app_) over 6 days with cascade blue, a standard fluorescent tracer for paracellular permeability in microfluidic models (*29*). *P*_app_ values remained stable between days 1 and 4, ranging from approximately 1.2 × 10^−6^ to 8.0 × 10^−7^ cm/s, indicating early formation of a functional barrier. By day 6, *P*_app_ decreased markedly, reflecting an enhanced barrier maturation over time (*30*, *31*) (Fig. 1I).

### Hormonal response to epithelial receptivity and stromal decidualization

**Fig. 2.**
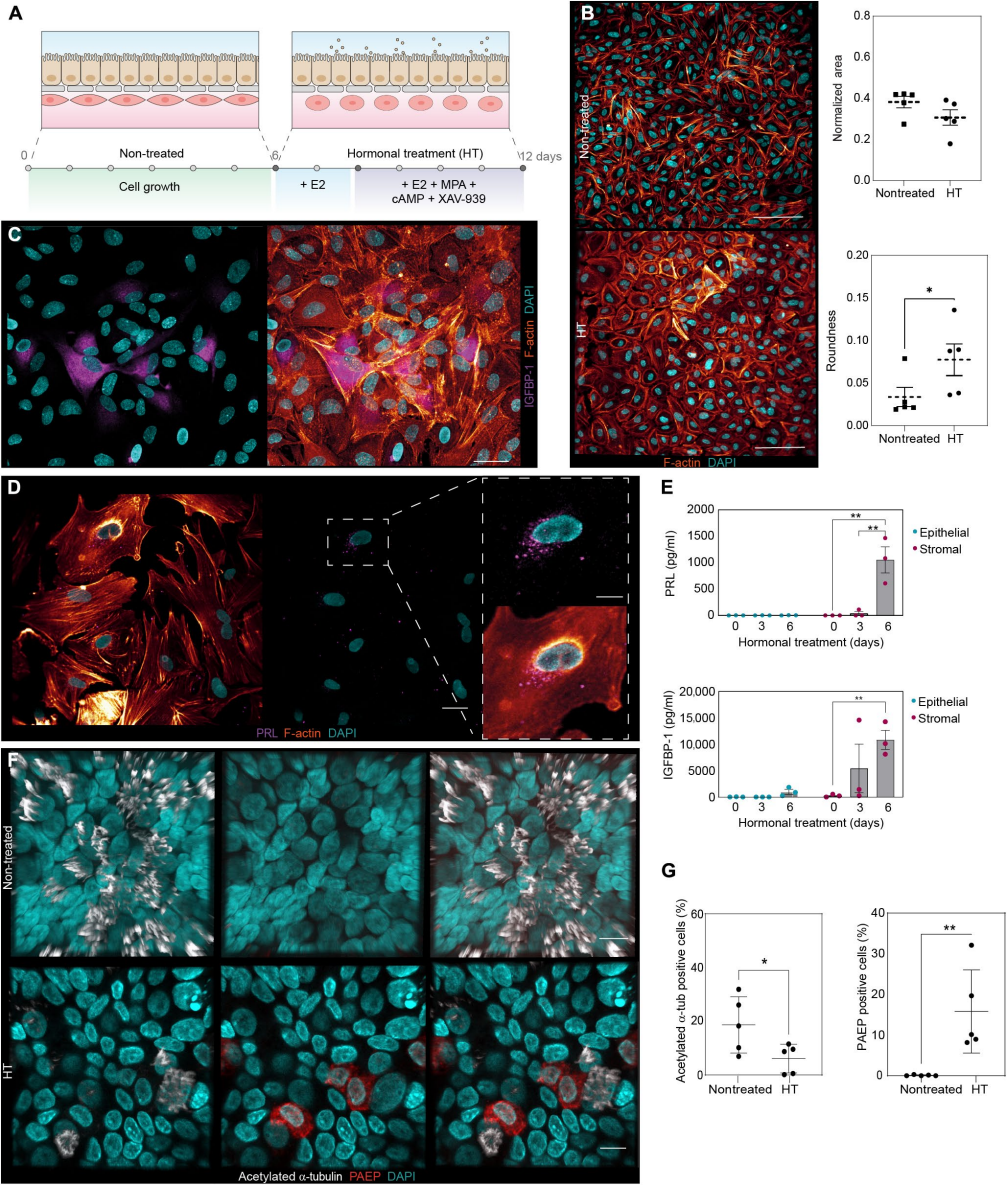
Epithelial receptivity and stromal decidualization in ADOC. (**A**) Schematic of the experimental setup. (**B**) Decidualization of stromal cells after 6 days of hormonal treatment. Left: Immunostaining of stromal cells before and after hormonal treatment with F-actin (orange) and DAPI (blue). Right: Morphological quantitative analysis showing increased roundness (top panel) and cellular area (bottom panel) of stromal cells after hormonal treatment. Data represent the quantification of five chips from three independent donors (roundness *P < 0.05 by Mann-Whitney test). Scale bars, 70 μm. (**C** and **D**) Presence of IGFBP-1 (purple) and PRL (pink) in response to hormonal treatment, along with DAPI (blue) and F-actin (orange) staining. Scale bars, 50 μm. (**E**) PRL and IGFBP-1 quantification using ELISA at days 0, 3, and 6 of hormonal treatment. Data are presented as mean ± SD, *n* = 3 independent chips from 3 different donors, **P < 0.01 by Bonferroni’s multiple comparisons two-way ANOVA. (**F**) Acetylated α-tubulin (white) and PAEP (red) in EECs before and after hormonal treatment. Scale bars, 10 μm. (**G**) Quantification of the percentage positive α-acetylated tubulin and PAEP cells before and after hormonal treatment. Data are presented as mean ± SD, of five chips from three independent donors (*n* = 5), **P < 0.01, Mann-Whitney test for PAEP and *P < 0.05 by unpaired *t* test for acetylated α-tubulin. All data were tested Shapiro-Wilk normality tests before running parametric or nonparametric tests.

To investigate ADOC functionality, we assessed hormone response to epithelial receptivity and decidualization. After 6 days of culture, both channels of ADOC were treated with 17β-estradiol (E2) for 2 days, followed by a combination of E2, medroxyprogesterone acetate (MPA), cyclic adenosine monophosphate (cAMP) and the Tankyrase Inhibitor XAV-939 for an additional 4 days (Fig. 2A and Materials and Methods). These components facilitate stromal decidualization while inhibiting the WNT/β-catenin pathway to create a receptive epithelium (*14*). In the stromal compartment, after 6 days of treatment, cells transitioned from a fibroblast-like to a more rounded morphology compared to unstimulated conditions (Fig. 2B), in agreement with the literature. Quantitative analysis revealed a tendency toward a decrease in cell area, although without significant differences (Fig. 2B). A significant increase in cell roundness was found when compared to nontreated cells, consistent with stromal decidualization remodeling (*32*) (Fig. 2B). Hormonal treatment also induced the secretion of Insulin-like Growth Factor-Binding Protein 1 (IGFBP-1) and prolactin (PRL), accompanied by progesterone receptor (PGR) expression, suggesting a functional hormone response and decidualization (Fig. 2, C and D, and fig. S2A). Enzyme-linked immunosorbent assay (ELISA) for PRL and IGFBP-1 performed with the culture supernatant during hormonal treatment showed no detectable levels on day 0, an increase by day 3, and a further rise by day 6 (Fig. 2E).

The upper epithelial monolayer also responded to hormonal stimulation, as evidenced by a reduction in acetylated α-tubulin–positive ciliated cells (18.67 ± 10.49% versus 6.10 ± 5.35%) and an increase in progestagen-associated endometrial protein (PAEP)/glycodelin-positive cells after treatment (0.9 ± 0.14% versus 15.81 ± 10.21%) (Fig. 2, F and G). In addition, PAEP was also significantly increased after full hormonal treatment (day 6; *P* = 0.0029, fig. S2B) as analyzed by ELISA. The hormonal treatment also affected the permeability barrier in the epithelial compartment, as evidenced by an increase in *P*_app_, which is consistent with the permeability changes previously described (*33*, *34*) (fig. S2C). These findings suggest that the upper epithelial channel becomes receptive and that ADOC replicates aspects of the secretory endometrium.

### Hormonal stimulation drives transcriptional changes in ADOC

**Fig. 3.**
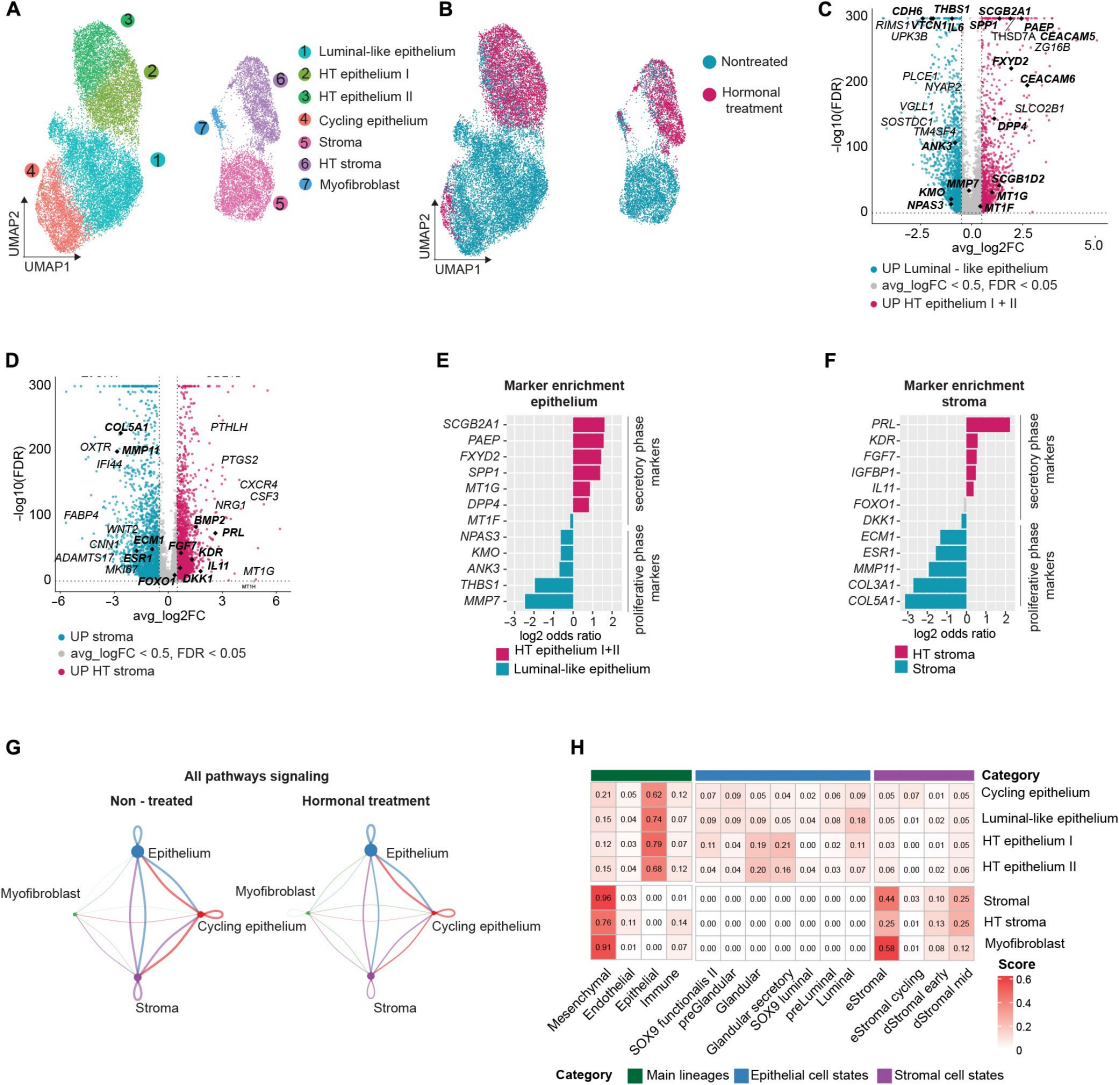
Single-cell transcriptomic profiling of ADOC. (**A**) UMAP visualization of single-cell integration of nontreated and treated cells showing all cell types identified in ADOC. (**B**) UMAP representation colored by hormone condition in ADOC. (**C**) Volcano plot showing differentially expressed genes (DEGs) between luminal-like epithelium and HT epithelium I + II. Genes in bold represent markers previously reported in human endometrial atlases (*35*, *36*). (**D**) Volcano plot showing DEGs between stroma and HT stroma. Genes in bold represent markers previously reported in human endometrial atlases (*35*, *36*, *39*). (**E**) Frequency-based enrichment of epithelial marker genes. Log2 odds ratios of marker-positive cell frequencies in HT epithelium versus luminal-like epithelium. Gene detection (UMI > 0) was compared using Fisher’s exact test; colors indicate significant enrichment after multiple testing correction. (**F**) Frequency-based enrichment of stromal marker genes. Log2 odds ratios of marker-positive cell frequencies in HT stroma versus stroma. Gene detection (UMI > 0) was compared using Fisher’s exact test; colors indicate significant enrichment after multiple testing correction. (**G**) Chord plots displaying the CCC network of all signaling pathways in nontreated and treated cells. (**H**) Cell type correspondence between in vitro and in vivo endometrial datasets. Heatmap showing the average prediction scores of in vitro cell types mapped to in vivo reference cell types (*35*). Rows represent in vitro cell types, and columns correspond to in vivo identities. The color scale indicates the mean prediction score. Category annotations group cell types into main lineages and epithelial or stromal subtypes. Groups with cumulative prediction scores above 0.10 are shown.

To further underpin the cell complexity of ADOC before and after hormonal treatment, we obtained high-quality transcriptomes from 28,212 individual cells using single-cell RNA sequencing (scRNA-seq; fig. S3, A and B). Cell clustering representation in the uniform manifold approximation and projection space (UMAP) as well as manual curation of canonical markers identified seven main cell types, including epithelial and stromal clusters, as well as a myofibroblast cell type (Fig. 3A and data S1). We observed that ADOC cells separated depending on their epithelial and stromal identity (Fig. 3A). In addition, cells mostly grouped depending on their hormonal treatment condition (Fig. 3B and fig. S3B). To determine the transcriptional effect of the hormonal treatment in ADOC we analyzed the differential expression between the luminal-like population (*VTCN1*, *IL6*, and *CDH6*) (data S1) detected mostly in nontreated chips, and two hormone-induced epithelial populations (HT I and HT II) mainly composed of treated cells (Fig. 3, A and B). We observed that the HT epithelium I and II presented high expression of secretoglobins (*SCGB2A1* and *SCGB1D2*) (*35*), glycoproteins (*CEACAM5* and *CEACAM6*) and glandular secretory markers (*PAEP*, *SPP1*, and *DPP4*) (Fig. 3C), resembling the acquisition of a secretory identity that could be recapitulating the human endometrium. In parallel, when we compared the nontreated stroma (*MMP11*, *ECM1*, and *COL3A1*) (data S1) with the HT stroma that appeared in ADOC upon HT, we observed an up-regulation of genes previously associated with decidual stroma and vascular development (*BMP2*, *DKK1*, *KDR*, and *IL11*) (Fig. 3D) (*35*–*37*).

To further characterize the cell identity of ADOC, we analyzed the expression frequency of marker genes characteristic of proliferative and secretory phases in vivo in our dataset (*36*, *38*, *39*). In the epithelial compartment, secretory markers [*PAEP*, osteopontin (*SPP1*), and Secretoglobin Family 2A Member 1 (*SCGB2A1*)], were significantly enriched in hormone-treated cells belonging to the HT epithelium I and II, whereas luminal markers were consistently enriched in the luminal-like epithelium (*THBS1* and *MMP7*) (Fig. 3E). In the stromal compartment, odds ratio analysis showed that classical decidualization markers, such as *PRL* and *IGFBP1*, were more frequently detected in HT stromal cells, although this trend was not observed for all decidual markers (Fig. 3F). In contrast, markers associated with proliferative stroma were more prevalent in the nontreated stroma cells (Fig. 3F), indicating that ADOC stromal cells initiate a differentiation program to decidualization following hormonal treatment (*36*, *38*). Over representation analysis further revealed the enrichment of pathways directly related to hormone response and metabolism in the HT epithelium I and II (fig. S3C) as well as in the HT stroma cluster (fig. S3D). Pathways associated with vascular endothelial growth factor production and angiogenesis were up-regulated in the stromal and epithelial populations after HT (fig. S3, C and D), suggesting that ADOC cells express factors involved in vascular development signaling, a critical feature of proper decidualization in vivo.

To demonstrate that ADOC exhibits a dynamic cross-talk between stromal and epithelial compartments, we performed a cell-cell communication analysis based on the expression of known ligands and receptors (*40*). This analysis revealed an active signaling between epithelial and stromal cells for both treatment groups (Fig. 3G), confirming the presence of a cross-talk between both compartments in ADOC. Notably, we observed that signaling pathways such as Bone Morphogenetic Protein (BMP) and SPP1, involved in blastocyst adhesion to the epithelium, stromal decidualization, and epithelial receptivity, were active in ADOC following hormonal treatment (fig. S3, E and F, respectively), thereby supporting the functional relevance of our model.

Last, we also compared our data with an in vivo endometrial scRNA-seq dataset (Fig. 3H and fig. S3G) (*35*). Although our model did not fully capture the complexity of all epithelial and stromal populations, the canonical correlation-based label transfer analysis showed that luminal-like cells had a high transcriptional similarity to in vivo luminal epithelium as shown in the marker analysis (Fig. 3H). The HT epithelium I and II preserved transcriptional similarity with glandular and glandular secretory clusters, reflecting the acquisition of a hormone-induced secretory phenotype. In the stromal compartment, the ADOC stromal cluster resembled the proliferative-phase endometrial stroma (eStromal) and had a high mesenchymal identity, whereas the HT stroma showed a loss in the mesenchymal ratio (Fig. 3H). These data revealed transcriptional shift toward a decidual phenotype, although the transition appeared less pronounced than in the epithelial cells. These results indicate that ADOC recapitulates the cellular composition of the endometrium in vivo.

### ADOC recapitulates endometrial extracellular vesicle secretion

**Fig. 4.**
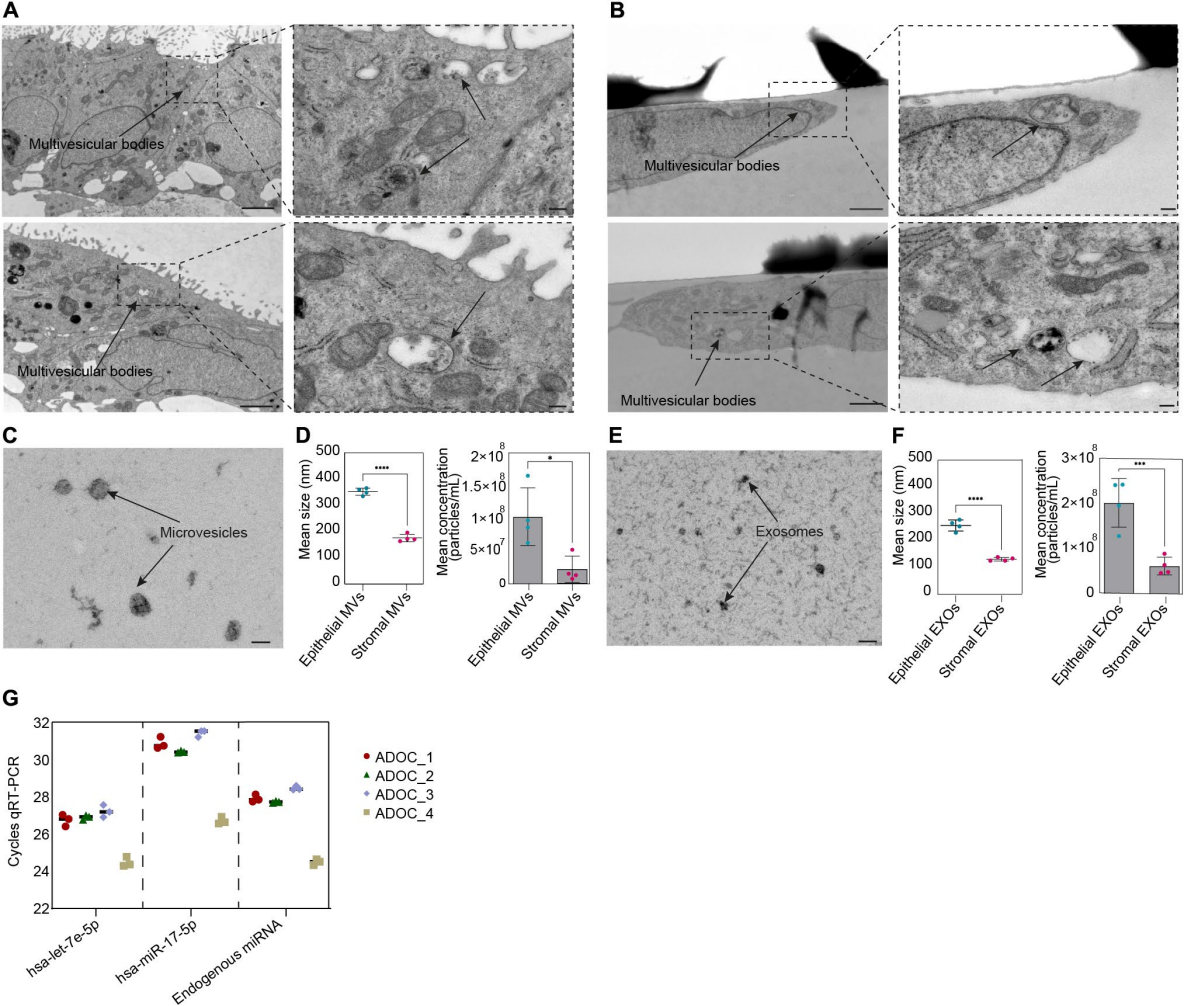
ADOC recapitulates endometrial EV secretion. (**A**) TEM micrographs of epithelial cells showing multivesicular bodies localized in the apical region of the cells. Scale bars, 2 μm (left) and 200 nm (right). (**B**) TEM micrographs of stromal cells displaying multivesicular bodies. Note that the empty spaces and black structures correspond to the membrane of ADOC. Scale bars, 1 μm (left) and 200 nm (right). (**C**) TEM micrograph of microvesicles (MVs) isolated from ADOC. Scale bar, 100 nm. (**D**) Nanoparticle tracking analysis (NTA) quantification of mean size (left) and concentration (right) of epithelial versus stromal MVs isolated from the epithelial and stromal compartments after 6 days of hormonal treatment (data are presented as mean ± SD, *n* = 4, ****P < 0.0001 and *P < 0.05, by unpaired *t* test). (**E**) TEM micrograph of exosomes (EXOs, arrows) isolated from ADOC. Scale bar, 100 nm. (**F**) NTA quantification of mean size (left) and concentration (right) of epithelial versus stromal EXOs isolated from the epithelial and stromal compartments after 6 days of hormonal treatment (data are presented as mean ± SD, *n* = 4 independent chips from three donors ****P < 0.0001 and ***P < 0.005, by unpaired *t* test). Scale bar, 500 nm. (**G**) RT-qPCR detection of selected microRNAs (let-7e-5p, miR-17-5p, and endogenous control) in epithelial-derived MVs isolated from four independent replicates (ADOC_1–4).

We next examined whether the model recapitulates the endometrial extracellular vesicle (EV) secretion, a process known to play a key role in maternofetal cross-talk (*41*–*43*). TEM micrographs revealed multivesicular bodies in epithelial (Fig. 4A) and stromal cells (Fig. 4B). To confirm EV secretion, we collected media from both compartments between days 5 and 11, which revealed the three major EV subtypes including apoptotic bodies (ABs), microvesicles (MVs), and exosomes (EXOs) (Fig. 4, C and E, and fig. S4A). To determine differences in the size and concentration of MVs and EXOs derived from both compartments, we performed nanoparticle tracking analysis (NTA). Our analysis revealed significant differences between epithelial and stromal derived MVs and EXOs (fig. S4, B and C). Specifically, the epithelial layer secreted larger EVs in greater quantities (Fig. 4, D and F, and fig. S4, B and C). Comparison of epithelial and stromal EVs before and after hormonal treatment showed no differences, suggesting that EV release is maintained independent of hormonal treatment (fig. S4D), in line with previous work (*43*). To test that in the secretory phase EVs resembled those in vivo, we extracted their miRNA content and analyzed the presence of endometrial-specific miRNAs including hsa-let-7e-5p and hsa-miR-17-5p typically enclosed within EVs during secretory phase, as reported in the literature (*44*). Quantitative reverse transcriptase polymerase chain reaction (PCR) analysis confirmed that endometrial-specific miRNAs were detected in ADOC EVs at cycle thresholds values comparable to ubiquitous EV miRNAs (Fig. 4G). This finding supports their presence in EVs produced by ADOC at physiologically relevant and detectable levels confirming that ADOC recapitulates the EV profile of the in vivo endometrium.

### Mouse embryos display adhesion in ADOC

**Fig. 5.**
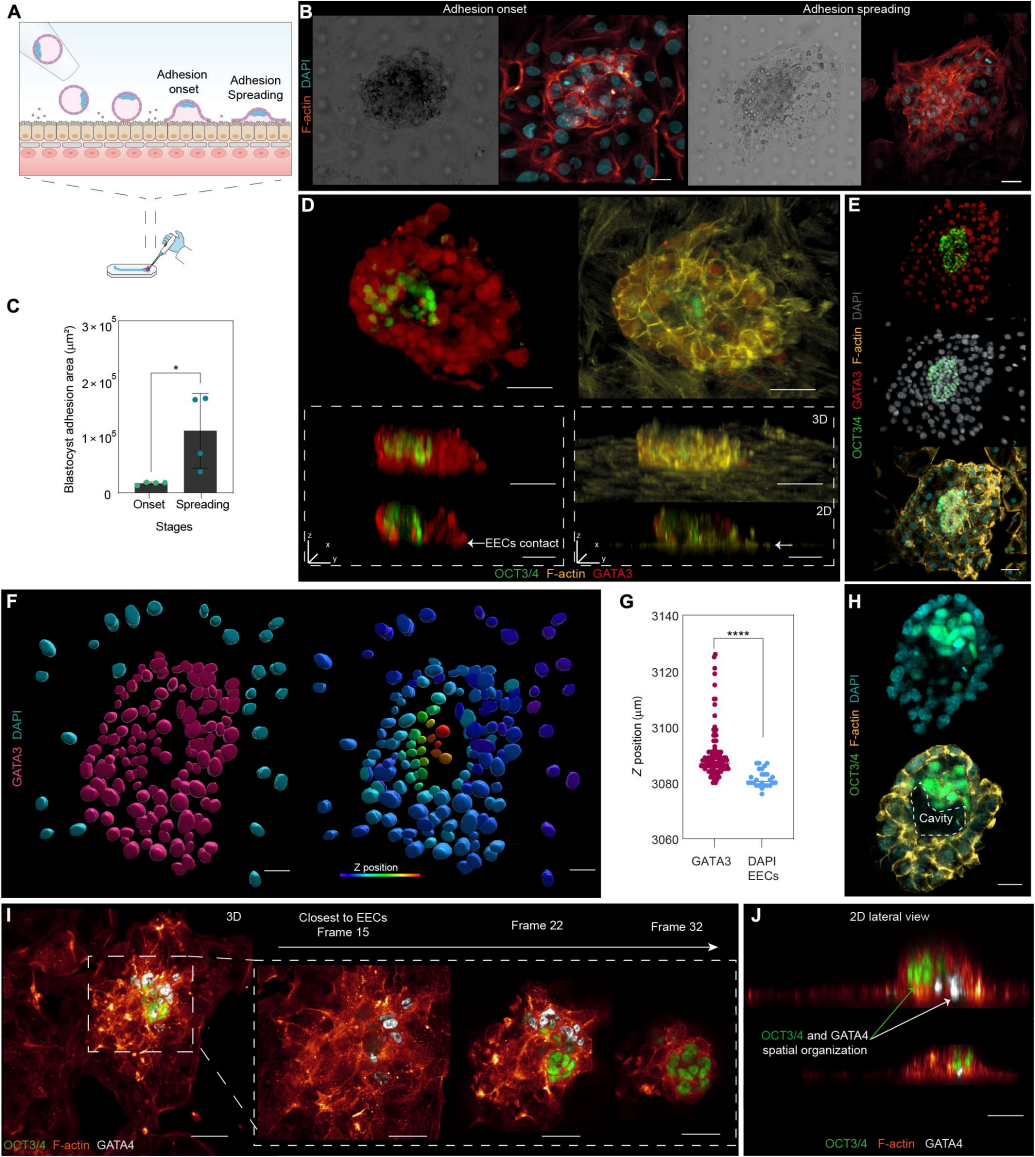
Mouse embryo adhesion confirms ADOC functionality. (**A**) Schematic of embryo positioning in ADOC from adhesion onset to spreading. (**B**) Mouse blastocysts during adhesion onset and spreading. Bright-field images (left) and immunofluorescence for F-actin (orange) and DAPI (blue) show changes in cell morphology and cytoskeletal organization. Scale bars, 40 μm. (**C**) Blastocyst area quantification during adhesion onset and spreading (mean ± SD, *n* = 8 embryos in four independent chips from four donors; unpaired *t* test, *P* < 0.05). (**D**) Immunofluorescence images (top) of adhered blastocysts showing inner cell mass (ICM; OCT3/4, green) and trophectoderm (TE; GATA3, red), with F-actin (yellow). Middle: 3D reconstruction of blastocyst adhesion in the epithelial compartment. Bottom: 2D *Y*-*Z* planes showing ICM (green) and TE (red). White arrows indicate TE contact with the epithelial layer. Scale bars, 50 μm (top), 80 μm (bottom, 3D), and 40 μm (bottom, 2D). (**E**) Fluorescent images of an expanded blastocyst and surrounding epithelial cells. OCT3/4 (green), GATA3 (red), F-actin (yellow), and DAPI (blue). Scale bar, 50 μm. (**F**) Computational segmentation of a spread embryo showing GATA3 nuclei relative to epithelial layers (DAPI EECs). Color scale indicates *Z* position: blue, closer to epithelium; red, farther. Scale bar, 30 μm. (**G**) Dot plot showing Z distribution of GATA3 and epithelial DAPI nuclei. (**H**) Immunofluorescence of adhered blastocysts showing ICM (green), DAPI (blue), and F-actin (yellow). Dotted lines indicate cavity presence. Scale bar, 20 μm. (**I**) 3D reconstruction and optical sections (frame 15 near epithelial cells; frames 22 and 32, upper) showing primitive endoderm cells (white) adjacent to the epithelial cell layer and apical epiblast cells (green). Scale bars, 50 μm (3D) and 40 μm (sections). (**J**) 2D lateral view showing apical OCT3/4 epiblast cells and GATA4 primitive endoderm cells (white) closer to the epithelial interface. Scale bar, 50 μm.

We then explored whether ADOC could be used to study embryo adhesion. Mouse blastocysts were used as a robust and well established model of early embryo-epithelium interactions, widely applied in heterologous adhesion assays (*45*–*47*), and served here as an initial validation tool to our assay conditions. Mouse blastocysts were introduced into the epithelial channel following hormonal treatment (Fig. 5A). A total of 23 mouse blastocysts successfully adhered to the epithelial compartment (52%, *n* = 44), confirmed by their resistance to media flushing (movie S1). Mouse embryo attachment progressed through an initial adhesion stage (“adhesion onset”), characterized by the firm attachment of the first embryonic cells to the epithelial layer, followed by the spreading of the embryo across the epithelium (Fig. 5, A and B). The average time at which adhesion occurred was 59.3 ± 29.5 hours (fig. S5A). During initial adhesion, the cells forming the embryo appeared densely packed while maintaining its spherical shape (Fig. 5B). Following this, the embryo gradually spread out over the epithelial surface, increasing contact area (“adhesion spreading”) (Fig. 5B). To quantify this morphological change, we measured embryo spreading area over the epithelial monolayer, revealing an increase in adhesion area compared to the onset of this process (Fig. 5C). Immunofluorescence for the trophectoderm (TE) marker GATA binding protein 3 (GATA3) confirmed that TE cells initiated contact, while the inner cell mass (ICM) labeled by octamer-binding transcription factor 3/4 (OCT3/4) remained apically positioned (Fig. 5D; fig. S5, B and C; and movie S2). In the spreading adhesion stage, we could confirm that TE cells spread and flattened over the epithelial layer (Fig. 5E), indicating that, following the adhesion onset, the trophoblast remains functional and could undergo the very early steps that precede invasion. To further characterize the spreading process, we performed computational analysis of TE nuclei positioning along the *Z* axis. This analysis confirmed that the majority of TE nuclei (GATA3) aligned within the same focal plane of the epithelium layer [4′,6-diamidino-2-phenylindole (DAPI) EECs] along the *Z* axis (Fig. 5F). This analysis provides quantitative evidence that TE nuclei reposition into the epithelial plane, consistent with full spreading (Fig. 5, F and G). Furthermore, a blastocoel cavity restructuring was observed (Fig. 5H and fig. S5D), suggesting ICM polarization. After full spreading, we also observed primitive endoderm reorganization, as revealed by the presence of GATA binding protein 4 (GATA4) localized closer to the EECs than OCT3/4 cells (Fig. 5, I and J, and fig. S5E).

### ADOC reveals adhesion dynamics and early lineage reorganization of human blastocysts

**Fig. 6.**
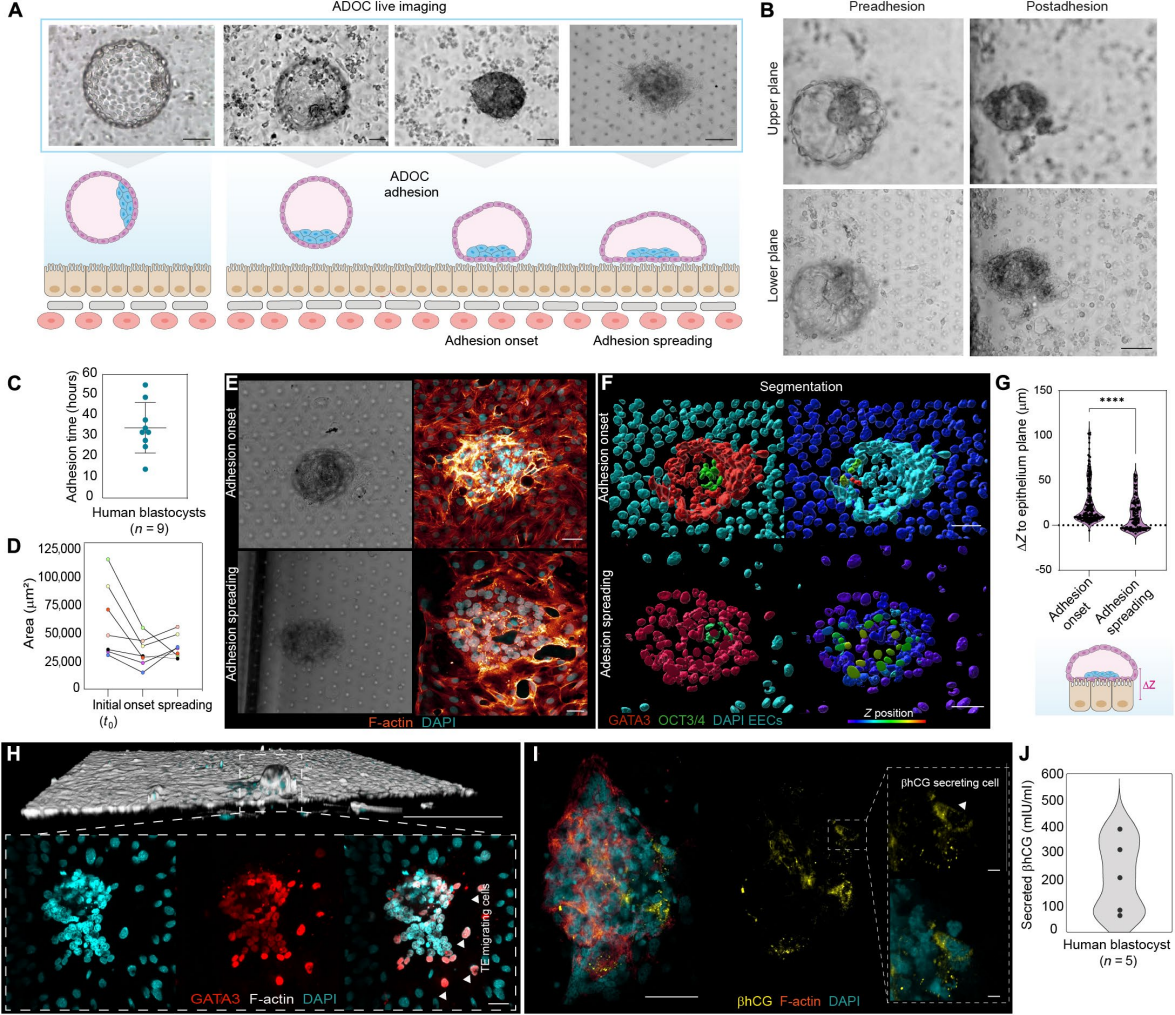
Human blastocyst adhesion dynamics in the ADOC model. (**A**) Time-lapse showing human blastocysts adhering to the epithelial layer in ADOC. Top: bright-field time-lapse images show morphological changes across adhesion stages. Bottom: schematic illustrate each stage observed in vitro. Scale bar, 50 μm. (**B**) Time-lapse images focusing on upper (embryo) and lower (epithelial cells) planes before (pre) and after (post) adhesion. Scale bar, 50 μm. (**C**) Average adhesion onset time for individual blastocysts (mean ± SD; *n* = 9 human embryos adhered in nine chips from four donors). Each dot represents one embryo. (**D**) Embryo area quantification in ADOC at three stages: initial placement (*t*_0_), onset of adhesion, and spreading. Each line represents a single embryo; colored points indicate temporal area evolution (seven of nine embryos quantified due to imaging limitations). (**E**) Immunofluorescence images of adhesion onset and spreading. Left: bright-field; right: F-actin staining (orange) reveals cytoskeletal reorganization. Scale bar, 50 μm. (**F**) 3D computational segmentation of embryos at adhesion onset and spreading. GATA3 (red) marks TE, OCT3/4 (green) the ICM and DAPI (blue) the epithelium. (**G**) Quantification of embryo position relative to the epithelial layer. Violin plots show *Z* distance (Δ*Z*, μm) between the embryo centroid and epithelial average reference plane during adhesion onset and spreading. Positive values indicate displacement above the epithelium (dots indicate individual nuclei, ****P < 0.0001, Mann-Whitney *U* test). Bottom schematic illustrates Δ*Z* relative to epithelial monolayer. (**H**) 3D side view of an adhered embryo during spreading. Zoom-in reveals TE (GATA3, red) extending along the epithelial surface, migrating laterally (arrowheads). Scale bars, 3D:100 and 50 μm. (**I**) Immunofluorescence showing βhGC release after implantation. Scale bars, 50 and 10 μm (zoom). (**J**) βhCG secretion by individual human blastocysts (*n* = 5 embryos in five chips from four donors). Violin plot shows βhCG levels (mIU/ml), dots indicate individual embryos.

We then studied adhesion of human blastocysts (days 5 to 6 postfertilization) after ADOC hormonal treatment. Following the selection of hatched human blastocysts, we used time-lapse bright-field imaging to record the dynamic process of embryo positioning and adhesion. During the adhesion process, the embryo underwent a morphological transition from a spherical, expanded blastocyst to a flattened compacted structure (Fig. 6, A and B). Preadhesion images showed a well-defined blastocoel and rounded morphology across both planes, whereas postadhesion images revealed a loss of sphericity, increased optical density, and close contact with the epithelial surface in the lower plane (Fig. 6B and movie S3). Of 19 blastocysts, 47% (*n* = 9) attached over a mean period of 34.1 ± 12.2 hours (Fig. 6C). To assess whether hormonal treatment was needed for successful adhesion, we performed the same assay under hormone-free conditions (no E2/MPA/cAMP/XAV939; *n* = 12 blastocysts). In contrast to the hormone-primed setting, embryos exhibited cycles of expansion and collapse but failed to establish stable adhesion (movie S4). Over time, blastocysts showed signs of structural breakdown, including collapse and cellular fragmentation (fig. S6A). Moreover, upon medium flushing, all embryos were easily displaced from the epithelial surface, confirming the absence of firm attachment. To further validate our model and its responsiveness to pharmacological modulation, we performed a contraceptive assay by adding mifepristone, a progesterone receptor antagonist (10 μM) (fig. S6B and Materials and Methods). Under these conditions, only one of eight embryos (12.5%) achieved a stable attachment, whereas the remaining embryos failed to adhere and were displaced upon flushing. When comparing adhesion rates of HT, nontreated, and mifepristone conditions, hormonal treatment increased embryo adhesion compared to nontreated controls, while mifepristone markedly reduced adhesion efficiency, consistent with its known anti-implantation effect (fig. S6C) and highlighting the need of a hormonal receptive system to support embryo adhesion.

Quantitative tracking of embryo area over time showed a reduction associated with blastocyst collapse, a morphological change that indicated the onset of embryo adhesion (Fig. 6D). This was followed by an increase in embryo area in most cases, reflecting trophoblast spreading across the epithelial layer, as also observed with F-actin staining (Fig. 6, D and E). 3D reconstructions of segmented embryos illustrated the Z position of each nuclei, showing that at the onset of adhesion, most embryo cells (GATA3) were positioned above the epithelial nuclei, as identified by DAPI staining (and GATA3− cells) (Fig. 6F). By contrast, during spreading, TE shifted closer to the epithelial nuclei, indicating increased integration with the epithelial surface (Fig. 6F). Quantitative analysis confirmed that the onset of adhesion displayed larger Δ*Z* values, whereas the adhesion spreading exhibited lower Δ*Z* values (Fig. 6G). In addition, late spreading showed lateral migration of TE cells toward the epithelial layer. Although ADOC is designed to study embryo adhesion, this early migratory event could suggest the onset of embryo invasion (Fig. 6H).

To study functional TE activity, we demonstrated that adhered embryos released β-human chorionic gonadotropin (βhCG) confirming that adhesion was coupled with βhCG hormone secretion (Fig. 6, I and J, and fig. S6D). Immunofluorescence analysis showed that βhCG signal colocalized with GATA3+ cells (fig. S6D), supporting its trophoblast origin, while ELISA quantification confirmed βhCG secretion into the culture medium (Fig. 6J), recapitulating early events of embryo adhesion (*48*). In addition, attached embryos exhibited features consistent with trophoblast differentiation toward a syncytiotrophoblast-like phenotype with the presence of multinucleated cells (fig. S6E), suggesting the emergence of syncytial-like structures during early implantation.

**Fig. 7.**
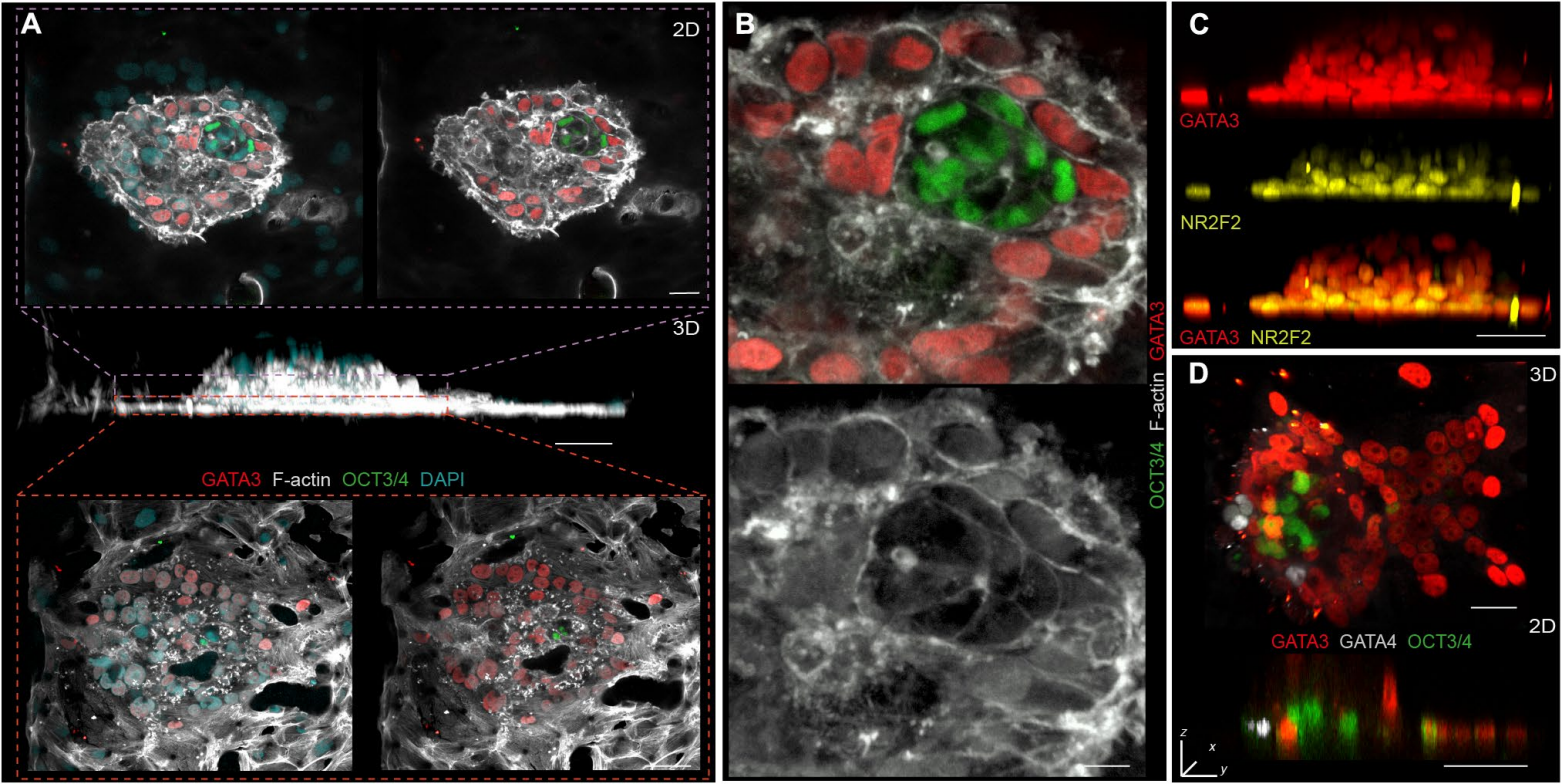
Human blastocyst adhesion in ADOC reveals TE spatial localization and ICM remodeling. (**A**) 3D projection of a blastocyst during adhesion. Zoom-in of optical sections at two different focal planes, one closer to the epithelial layer (below, orange dashed lines) and one farther away (above, purple dashed lines) demonstrating that few OCT3/4 cells positioned adjacent to the epithelial surface, while GATA3 TE cells occupy from basal to apical positions. Scale bars, 50 (3D) and 30 μm. (**B**) The cluster of OCT3/4 cells (green) representing the ICM are centrally located and surrounded by GATA3 cells (red). The OCT3/4 cells form a compact, radially organized structure, as shown with F-actin (white) Scale bar, 15 μm. (**C**) 3D side views of adhered blastocysts stained for OCT3/4 (green), GATA3 (red), and NR2F2 (yellow). Scale bars, 50 μm. (**D**) 3D projection showing three distinct embryonic lineages: GATA4 hypoblasts (white), OCT3/4 epiblast (green), and GATA3 TE (red). Scale bars, 30 and 50 μm.

We further explored spatial cell arrangement during human blastocyst adhesion. Optical sections from different planes revealed that the lower plane, adjacent to the epithelium, was primarily composed of GATA3 TE cells, but also included a few internally positioned OCT3/4 cells, all merged across the epithelial layer (Fig. 7A, below). In the upper plane, OCT3/4 cells were compactly clustered and surrounded by TE cells (Fig. 7A, above), suggesting that the ICM remained largely internal but still in close contact with the epithelial layer during the first steps of adhesion (Fig. 7A and movie S5). Higher magnification into the ICM (Fig. 7B) confirmed a radially organized OCT3/4 cell cluster surrounded by GATA3 cells, suggesting the first steps of lumen formation, indicative of embryo initial reorganization (*49*) (fig. S6F).

To confirm that embryos adhered via the polar TE, we examined the localization of nuclear receptor subfamily 2 group F member 2 (NR2F2), a marker of polar TE identity in the human blastocyst. NR2F2 cells were localized at the region of the TE in contact with the epithelial surface, confirming that embryo adhesion in ADOC occurs via the polar TE (Fig. 7C), thus supporting correct embryo orientation for adhesion and developmental progression. To study further lineage specification, staining for GATA4 revealed additional ICM complexity, with GATA4 hypoblast cells reorganizing with OCT3/4 epiblast and GATA3 TE populations (Fig. 7D). These three lineages were segregated in 2D orthogonal views demonstrating first stages of lineage specification. These sections showed that a layer of GATA4 and OCT3/4 cells were consistently positioned closer to the epithelial layer along with GATA3 cells, which surrounded the periphery and extended apically (Fig. 7D). Together, ADOC represents a novel microfluidic cell-based system that enables cell communication while supporting the study of human adhesion-associated features through live observation and molecular characterization in a controlled environment.

## DISCUSSION

Early stages of human implantation, including embryo adhesion to endometrium, remain poorly understood due to ethical constraints and the inherent challenges of accessing these events in vivo. Studies have focused on in vitro human endometrial models, which range from 2D primary tissue cultures to organoid-based and microfluidic systems. Although some of these models allow the coculture of human embryos or blastoids to support different implantation stages, they have not yet succeeded in capturing the precise timing of embryo adhesion or identifying the cellular hallmarks that characterize this critical phase (*50*). To address this, we developed ADOC, an endometrium-on-a-chip with a compartmentalized microarchitecture that recapitulates epithelial-stromal interactions and allows independent molecular analysis as well as selective treatment of each compartment. Morphological characterization of ADOC revealed a polarized columnar epithelial layer closely resembling the native endometrial architecture. At the molecular level, scRNA-seq identified up to seven epithelial and stromal populations and ADOC cells clustered depending on their hormonal status. This analysis demonstrates that upon hormonal treatment ADOC cells shift from a basal state toward a receptive phenotype. This transition was characterized by epithelial up-regulation of secretory markers (*PAEP* and *SPP1*), together with stromal decidualization, as evidenced by increased expression of *IGFBP-1* and *PRL* and enrichment of vascular-related pathways, essential to promote effective decidualization (*36*, *38*, *51*, *52*). Transcriptomic profiles in hormonal treated chips aligned with glandular epithelial and decidual stromal transcriptomic signatures in vivo, indicating that ADOC faithfully recapitulates the mid-secretory phase (*35*). In ADOC, the spatial organization of epithelial cells adjacent to decidualized stroma recreates the architecture and signaling cross-talk required for embryo adhesion. Consistent with this, our scRNA-seq cell-cell communication analysis identified progesterone-driven epithelial IHH signaling toward stromal COUP-TFII/BMP2, coordinating epithelial receptivity and decidualization (*53*, *54*), as well as activation of SPP1-mediated pathways to promote blastocyst attachment (*55*).

Building on this molecular framework, we next assessed whether ADOC could capture functional embryo-endometrium interactions. We visualized human blastocyst apposition and adhesion, including polar TE spreading and ICM repositioning under hormonal conditions. Previously 3D models (*15*, *16*) offered valuable insights into human implantation but were limited by imaging depth and architectural complexity, hindering spatial resolution of embryo-epithelium interactions. Whilst initiation of adhesion by the polar TE has been reported in vitro (*56*), our model uniquely enabled visualization of this process alongside with ICM radial organization, resembling early lumenogenesis (*56*, *57*). Furthermore, we demonstrated reorganization of hypoblast-like cells during adhesion spreading which aligns with previous in vitro studies of embryo differentiation and implantation assays using blastoids (*14*, *16*).

Despite these strengths, this study presents some limitations. First, the porous membrane within the chip acts as a physical barrier, which represents a simplification of the native endometrial architecture, where the stromal compartment is a thick, heterogeneous tissue and where epithelial-stromal cells are directly interconnected. Because of its architecture, ADOC has just been used to study embryo adhesion. However, the chip could be redesigned to include more interconnected channels, hydrogels, or the integration of other cell types (*19*, *23*). Second, polydimethylsiloxane is known to absorb hydrophobic molecules such as steroid hormones that may lower the effective hormone dose. Although continuous perfusion and identification of decidualization markers confirmed that hormone availability was not limited in our system, it needs to be taken into consideration. Third, even if early embryo-epithelium interactions are partially conserved across species, implantation remains highly species-specific. Therefore, the use of mouse blastocysts in this model could be seen as a limitation, as adhesion to the human endometrial cells reflects endometrial permissiveness rather than fully species-matched implantation. In this matter, the use of embryo surrogates, such as blastoids, could have been more appropriate to assess chip validation. Nonetheless, heterologous adhesion assays have been used before providing a robust and well-established system for assay calibration (*45*–*47*). This limitation was mitigated by the inclusion of human embryos, which highlights the physiological relevance of ADOC.

In conclusion, ADOC current design reproduces the cellular hallmarks and paracrine signaling pathways between epithelial-stromal cells of the endometrium. This system has allowed us to demonstrate critical aspects of human embryo adhesion by live imaging and precise molecular dissection of embryo adhesion. ADOC provides a physiologically relevant platform for functional genomics, mechanistic assays and pharmacological screenings to identify compounds that could improve embryo adhesion impairment. Given that endometrial diseases and implantation failure remain a major challenge in assisted reproduction (*58*, *59*), ADOC offers a powerful tool to investigate the mechanisms underlying these pathologies.

## MATERIALS AND METHODS

### Experimental design

A commercial microfluidic device (Emulate Inc., Boston, USA) with two interconnected channels was used to develop an endometrium-on-a-chip model for studying embryo adhesion. Endometrial biopsies were collected from patients under ethical approval, and primary cells were processed to separate epithelial and stromal fractions. The epithelial cells were cultured as organoids, while stromal cells were maintained in a 2D culture system. The organoids were dissociated and seeded into the upper channel of the microfluidic device to create a monolayer, while stromal cells were seeded into the lower channel. Both cell types were expanded under continuous flow for 6 days, with organoid and stromal medium, respectively. To mimic the secretory phase of the endometrium, the model was treated with E2 for 2 days, followed by a combination of E2, cAMP MPA, and XAV939 for 4 days.

To test the functionality of the model, mouse blastocysts (*n* = 44) were devitrified, assisted-hatched, and cultured overnight in G2 plus medium. Two hours before implantation, the blastocysts were transferred to EmbryoGlue and placed in the epithelial channel of the device, where adhesion was monitored using confocal microscopy with time-lapse imaging. To study human adhesion dynamics, human blastocysts (*n* = 39; days 5 to 6 postfertilization) donated for research were devitrified, assisted-hatched, and cultured for 2 hours in embryo medium before being transferred to EmbryoGlue and cultured overnight. The human embryos were then introduced into the epithelial channel and monitored for adhesion events using confocal microscopy.

Mouse and human embryos were fixed at different stages of adhesion and characterized using immunofluorescence to evaluate molecular markers and adhesion progression. This experimental design allowed the assessment of the model’s ability to support and study embryo adhesion under conditions that simulate the human endometrium.

### Samples and ethical approvals

All tissue samples used in this study were obtained with written informed consent from all participants, in full compliance with the ethical guidelines set forth in the Declaration of Helsinki 2000. Superficial endometrial biopsies were collected as part of the IGX1-ORG-FV-21-01 project, which was approved by the Clinical Research Ethics Committees of Clínica El Pilar (Barcelona) and Hospital Clínico Universitario de Valencia. Eligibility criteria for participation in the study included female patients aged 18 to 42 years, within the reproductive age range, with a natural menstrual cycle and a body mass index between 18.5 and 29.9 kg/m^2^. Participants with oncological conditions, intrauterine device use within the past 3 months, hormonal treatment within the previous month, or bacterial or viral infections were excluded from the study.

Human embryos donated for research as surplus from IVF treatments were obtained from Next Fertility clinics. Their use was approved by the Ethics Committee on Drug Research of the Hospital Universitario y Politécnico La Fe (Valencia), as well as by the National Commission on Assisted Human Reproduction and the Commission on Guarantees for the Donation and Use of Human Tissues and Cells (Spain), under the framework of project FCS-IMA-CS-22-2.

### Processing of endometrial biopsies

A two-stage dissociation protocol was used to desegregate endometrial biopsies into stromal fibroblast and epithelial-enriched single-cell suspensions (*36*). First, the sample was rinsed with phosphate-buffered saline (PBS) (Biowest, L0615) in a petri dish to remove blood and mucus. Then, the tissue was minced into small pieces and dissociated with collagenase V (Sigma-Aldrich, C9263), RPMI (Gibco, 21875-034), 10% fetal bovine serum (FBS, Biowest, S181B-500), and deoxyribonuclease (DNAse) I (Sigma-Aldrich, 11284932001) at 37°C for 25 min under continuous shaking (175 rpm). The content was transferred to falcon through a 100-μm cell strainer filter. The tissue remaining on the filter was used for epithelial enrichment by incubating with 10-ml trypsin–DNAse I for 10 min in the previous conditions. The resulting two contents were transferred to 50 ml with 20 mL RPMI and filtered with 100-μm cell strainers.

### Establishment and culture of EEOs

EEOs were generated using previous described protocol (*11*) with minor modifications. The epithelial fraction obtained from the biopsies was resuspended in 30% Dulbecco’s modified Eagle’s medium/Ham’s F12 (DMEM/F12; Thermo Fisher Scientific, 11330032) and 70% Matrigel (Corning, 356231) and supplemented with the rho-associated protein kinase, Rock inhibitor (RI; Y-27632, Merck SCM075), at a final concentration of 2 μM. The suspension was cultured in 20-μl droplet deposited in prewarmed 48-well plates. Organoids were cultured as previously described (see table S1) (*60*). Matrigel droplets were cultured for 15 days in the first passage (P0), and for 7 days in the following passages. The medium was changed every 2 days. Organoids were recovered for passaging by liquifying Matrigel droplets with ice-cold DMEM/F12. Then, the organoids were dissociated using TrypLE Select (Gibco, 12563-029) for 7 min at 37°C supplemented with 1 μl of RI and mechanically triturated. The resulting cells suspension was centrifugated at 300*g*, resuspended in 70% Matrigel and 30% DMEM, for further culture or downstream applications.

### ESCs culture

The stromal fraction was seeded in T-25 flasks using DMEM-F12-Glutamax (Gibco, 10565) media supplemented with 10% FBS, 0.2% gentamycin (Gibco, 15750037), and 0.2% amphotericin B (Cultek, 5530-003-CF) for expansion and selection of fibroblastic-like cells. The cells used were from early passages (P0 to P5).

### Human preimplantation embryos culture

Human donated embryos (days 5 to 6 postfertilization) were thawed using Cryotop safety thawing kit following the manufacturer’s instructions (Kitazato, VT602) and underwent a 30% of assisted hatching (Octax Eyeware Laser, Vitrolife). Embryos were cultured in 30-μl droplet of embryo medium (*16*) (table S2) covered with Hypure oil light (Kitazato, 96005) for 2 hours, then embryos were cultured in EmbryoGlue (Vitrolife, 10085) overnight at 37°C and 5% CO_2_.

### Mouse embryos culture

Mouse blastocysts (Embryotools, PRO-005) were thawed following manufacturer’s instructions (Cryotop safety thawing kit, Kitazato, VT602) and underwent a 30% of assisted hatching. Blastocysts were cultured in 30 μl of G2 plus (Vitrolife, 10132) droplets cover with Hypure oil light overnight at 37°C and 5% CO_2_. Then, blastocysts were transferred to EmbryoGlue for 2 hours before its placement in the upper channel of the chip.

### Establishment of human endometrium-on-a-chip

The ADOC model is based on a commercially available platform (Emulate, Emulate Inc., Boston, USA), in which we culture EEOs cells and primary stromal endometrial cells. The chip was activated with Emulate proprietary reagents ER-1 (Emulate, 10461) and ER-2 (Emulate, 10462), which were mixed to reach a 1 mg/ml concentration and added to the top and bottom microfluidic channels. Subsequently, the chip was irradiated with ultraviolet light at a wavelength of 365 nm (NailStar, NS-01-EU) for 20 min. Then, both channels were coated with tissue-specific ECM components, collagen type I (100 μg/ml; Merck, C3867-1VL), and fibronectin (25 μg/ml; Merck, 11080938001) for stromal cells, and Matrigel (100 μg/ml; Corning, 356231) for epithelial cells. The coated chip was incubated 2 hours in the incubator at 37°C followed by a 4°C overnight incubation. The next day, EEOs were disaggregated, and single epithelial cells were seeded (1 × 10^5^ cells/35-μl confluence) on the upper channel. By deriving epithelial cells from EEOs rather than isolated primary cells, we ensure the cells retained proliferative capacity, viability, and polarity. The following day, ESCs were seeded (1 × 10^5^ cells/15-μl confluence) on the bottom channel. To promote growth of stromal cells in contact with the underside of the membrane, the chips were immediately inverted after cell seeding. Chips were connected to the Zoë instrument and perfused continuously at a flow rate of 30 μl/hour. On the basis of the device geometry, this corresponded to an estimated shear stress of 0.003 dyn/cm^2^ in the epithelial (top) channel and 0.09 dyn/cm^2^ in the stromal (bottom) channel. The media used to grow cells before hormonal treatment (expansion media, EM) consisting in organoid and stromal media, respectively. The cells were with EM for 6 days.

### Hormonal treatment of endometrium on chip

After culturing the epithelial cells and the stromal cells for 6 days in expansion medium, the hormonal treatment was initiated. First, the cells were treated for 2 days with 10 nM E2 (Sigma-Aldrich, E2758) and followed by 4 days of treatment with a combination of 10 nM E2, 1 μM MPA (Sigma-Aldrich, M1629), 250 μM cAMP (Sigma-Aldrich, B7880), and 10 μM XAV939 (Deltaclon, S1180) for 4 days to mimic the secretory phase.

### Contraceptive assay

Mifepristone treatment was performed following the standard hormonal treatment protocol. After 6 days of hormonal treatment with E2, MPA, cAMP, and XAV-939, mifepristone (10 μM) was added to the culture medium and maintained under continuous perfusion for 24 hours.

Subsequently, both epithelial and stromal channels were rinsed with 200 μl of EmbryoGlue and incubated for at least 2 hours at 37°C before human embryo placement.

### In vitro implantation assay

After 6 days of hormonal treatment, ADOC epithelium was prepared at least 2 hours before the placement of blastocysts by washing the channel two times with 200 μl of EmbryoGlue and kept at 37°C. The embryos were transferred to the upper channel using a 200-μl pipette in Embryoglue mimicking clinics transfer under an inverted microscope. The chips were placed over an Ibidi plate (Ibidi, 82107) to perform live imaging. To confirm embryo adhesion, PBS was flushed into the upper channel of the chip. Embryos that remained adhered following the flushing were used for subsequent analyses.

### Immunofluorescence of ADOC

First, upper and down channels were washed with 200 μl of PBS three times, and then cells were fixed with 4% paraformaldehyde (Thermo Fisher Scientific, 28908) for 30 min at room temperature. After fixing, the channel was washed three times with PBS. The cells were permeabilized in PBS 0.5% or 1% Tween-20 (Sigma-Aldrich, P9616) between 1 and 3 hours, and block with PBS 0.1% Tween-20 5% bovine serum albumin (BSA; Myltenic Biotec, 130-09-376) for 1 hour, all the steps were performed at room temperature in agitation. The samples were then incubated overnight at 4°C in agitation with primary antibodies (table S3) diluted in blocking solution. The day after, the samples were washed with PBS 0.1% Tween-20 at least three times. The washing buffer was replaced by the secondary antibodies (Invitrogen) (table S4) diluted in blocking buffer and incubated between 2 and 3 hours at room temperature in agitation. Then, the samples were washed at least three times with PBS 0.1% Tween-20. To finish a last incubation with DAPI (Merck, D9542) and/or phalloidin (ab235137, ab176759, Abcam; A34055, Invitrogen) diluted in PBS-BSA 1% was performed between 30 min and 1 hour. Last, the channel was washed with PBS for three times.

### Immunofluorescence of tissue sections

Paraffin-embedded tissue sections were incubated overnight at 60°C to facilitate wax melting and improve tissue adherence. Sections were rehydrated by sequential incubation in xylene (2×), followed by graded ethanol (100, 90, 70, 50, and 30%) and lastly rinsed in ddH_2_O. Samples were treated using antigen retrieval buffer (Abcam, ab93678) boiling in a pressure cooker for 20 min. After cooling, a photobleaching step was carried out by immersing the samples solution of NaOH 26.4 mM and 4.5% H_2_O_2_ in PBS, followed by exposure to a high-intensity light-emitting diode light source for 45 min. This step was repeated twice, and then samples were washed four times in PBS for 3 min.

Samples were incubated in blocking buffer (5% BSA in PBS–Tween 0.1%) for 30 min at room temperature, then primary antibodies were diluted in antibody solution (5% BSA in PBS–Tween 0.1%) and the samples were incubated overnight at 4°C. The next day, sections were washed three times in 0.1% Tween–PBS for 15 min and re-block for 30 min at room temperature before incubation with secondary antibodies diluted in antibody solution for 90 min at room temperature. Last, three washed for 15 min with 0.1% Tween–PBS were carried out. The samples were mounting with Prolong diamond with DAPI (Thermo Fisher Scientific, P36962).

### Transmission electron microscopy

For electron microscopy studies, samples were fixed with 3% glutaraldehyde in 0.1 M phosphate buffer, and membranes were extracted from the chip. Samples were postfixed with 2% osmium, rinsed, dehydrated, and embedded in Durcupan resin (Fluka, Sigma-Aldrich, St. Louis, USA). Ultrathin sections (0.06 to 0.08 μm) were made with an Ultracut UC-6 (Leica Microsystems, Wetzlar, Germany) with a diamond knife, stained with lead citrate (Reynold’s solution), and examined under a transmission electron microscope FEI Tecnai G2 Spirit BioTwin (Thermo Fisher Scientific, Oregon, USA). All images were acquired with a Xarosa digital camera (EMSIS GmbH, Münster, Germany) controlled by Radius software (version 2.1).

### ELISA assays

#### IGFBP-1 and PRL detection

Conditioned media from the top and bottom outlets were collected on days 0, 3, and 6 of hormonal treatment. PRL (Boster Biological Technology, EK0593) and IGFBP-1 (Raybiotech, ELH-IGFBP1-1) concentrations were assayed using commercial ELISA kits according to the manufacturer’s instructions. A 1:20 dilution of the media was used for testing IGFBP-1 and a dilution 1:2 of the media was used for tested PRL. The IGFBP-1 test limits detection to 5 pg/ml and the PRL test to 10 pg/ml. Each test was quantified by measuring optical density at 450 nm in a Multiscan Go (Thermo Fisher Scientific) concentrations were calculated from a standard curve generated for each assay.

#### PAEP detection

Culture medium from the epithelial channel was collected on days 2 and 6 of hormonal treatment. PAEP concentrations were quantified using a human PAEP ELISA kit (Abcam, ab275904) according to the manufacturer’s instructions. Medium samples were assayed at dilutions of 1:100, 1:200, and 1:1000. The assay sensitivity was 3 pg/ml. Optical density was measured at 450 nm using a MultiScan Go (Thermo Fisher Scientific), and PAEP concentrations were calculated from a standard curve generated for each assay.

#### βhCG detection

The media from epithelial channels were collected at various time points following human embryo adhesion and stored at −20°C until use. The medium was tested for βhCG levels using βhCG ELISA (Abcam, ab1786339) according to manufacturer’s instructions, alongside with βhCG standards. The results were quantified by measuring optical density at 450 nm in Multiscan Go (Thermo Fisher Scientific) and concentrations were calculated from a standard curve generated for each assay.

### Permeability assay

To evaluate the permeability of the epithelial barrier formed in the chip we added Dextran Cascade Blue 3000 MW (Thermo Fisher Scientific) (*61*) to the top channel inlet reservoir at 100 μg/ml diluted in expansion organoid media using a flow rate of 30 μl/hour. Culture media from the top and bottom inlet reservoirs and top and bottom outlet reservoirs were sampled every 24 hours after chip connection to the Zoë perfusion system. The fluorescence intensity of Cascade Blue was measured at 400/420 nm excitation/emission wavelengths using a Victor III plate reader (PerkinElmer Inc., Waltham, USA).

The apparent paracellular permeability (*P*_app_) was calculated on the basis of a standard curve using the following formula

$$P_{\text{app}}\left(\frac{\text{cm}}{s}\right)=\frac{C_{\text{output}}\left(\frac{\text{mg}}{\text{ml}}\right)\times \text{Flow rate}\;\left(\frac{\text{ml}}{s}\right)}{C_{\text{input}}\left(\frac{\text{mg}}{\text{ml}}\right)\times A\;\left({\text{cm}}^{2}\right)}$$

where *C*_output_ is the concentration of Cascade Blue in the effluents of the stromal compartment, *A* is the seeded area of epithelial channel, and *C*_input_ is the input concentration of dextran spiked into the epithelial compartment.

### Extracellular vesicle isolation

EVs were isolated from conditioned media collected from top and bottom outlet reservoirs in nontreated and hormonal treated chips. Stromal culture media supplemented with 10% FBS contains a variable amount of small EVs; therefore, EVs were removed from FBS by ultracentrifugation at 120,000*g* for 70 min at 4°C using a P50AT4 rotor in a CP100NX centrifuge (Hitachi) to ensure the isolation of only EVs from stromal cells within the chip. The supernatant containing EV-free FBS was used to supplement the stromal media for this experiment. Isolation of EVs from effluent culture media was performed as previously described with minimal modifications (*42*). Culture medium was centrifuged at 300*g* for 10 min to pellet residual cells and debris. Supernatants were centrifuged at 2000*g* for 10 min to separate and pellet the AB fraction. Pellets were resuspended in 1 ml of cold PBS and centrifuged at 2000*g* for 10 min to obtain a clean AB fraction. Supernatants were passed through a 0.8-μm-diameter filter (Whatman plc Maidstone) and centrifuged at 10,000*g* for 40 min to pellet the fraction enriched in MVs. Pellets were resuspended in 1 ml of cold PBS and centrifuged at 10,000*g* for 40 min to obtain a clean MV fraction. Last, supernatants were passed through 0.22-μm-diameter filters (Acrodisc syringe filters, Pall Corporation) and centrifuged at 120,000*g* for 70 min to pellet the EXO fraction of the media. Pellets were resuspended in 1 ml of PBS and centrifuged at 120,000*g* for 70 min to obtain a clean EXO-enriched fraction. All centrifugation steps were conducted at 4°C, and pellets for ABs, MVs, and EXOs after the PBS centrifugation step were resuspended in 50 μl of PBS and saved at −80°C.

### NTA of isolated EVs

EV size distribution and concentration in the effluent media of chips were measured by NTA principles in a NanoSight 300 instrument (Malvern Instruments Corp.). Because of the limited size working range (<1000-nm particle), ABs could not be analyzed. Ten microliters of the resuspended pellet of EVs were diluted in 700 μl of PBS and introduced into the NanoSight 300. The starting media volume for EV isolation was used to normalize concentration measurements.

### miRNA qPCR analysis

miRNA isolation from MVs obtained from collected media was performed using the miRNeasy micro kit (Qiagen, 217084) following the manufacturer’s instructions. For the isolation the ath-miR159a (5′phos-UUUGGAUUGAAGGGAGCUCUA-3′; IDT) was spike-in as exogenous control. Total isolated RNA was quantified using the QuickDrop instrument (Molecular Devices).

Complementary DNA (cDNA) synthesis of miRNAs was performed using the TaqMan Advanced miRNA cDNA Synthesis Kit (Thermo Fisher Scientific, A28007) from 2 μl of total isolated RNA following the manufacturer’s instructions. Real-time qPCR of miRNAs was performed using TaqMan Fast Advanced Master Mix (Thermo Fisher Scientific, 4444557) and specific probes for each miRNA (Thermo Fisher Scientific) in a total volume reaction of 10 μl in a 384-well-plate and a QuantStudio 5 Real-Time PCR System (Thermo Fisher Scientific).

The expression of hsa-let-7e-5p (A25576, 478579_mir) and hsa-miR-17-5p (A25576, 478447_mir) was evaluated. The miRNAs hsa-miR-200c-3p (A25576, 478351_mir) and hsa-miR-92a-3 (A25576, 477827_mir) were used as endogenous controls. To normalize data, we used exogenous controls. To evaluate the expression of miRNAs in the MVs, we compared the cycle threshold (Ct) values of the target miRNAs to those of ubiquitously expressed endogenous controls.

### scRNA-seq library preparation and sequencing

For single-cell transcriptomic analysis, stromal and epithelial cells from control and hormone-treated ADOC were collected separately. Stromal cells were first detached by incubation with TrypLE Select for 15 min and recovered in stromal medium. Subsequently, epithelial cells were dissociated by a 30-min incubation with TrypLE Select and recovered in organoid medium. Both cell fractions were centrifuged at 300*g* for 5 min at room temperature, the supernatant discarded, and the pellet resuspended in PBS supplemented with 0.04% BSA. Cell concentration and viability were determined using a LUNA-FL dual fluorescence cell counter (Logos Biosystems) with acridine orange/propidium Iodide staining (Logos Biosystems, F23001).

Approximately 17,000 cells (combined stromal and epithelial fractions) were loaded onto 10X Chromium G Chip (10X Genomics, PN-10000204) to generate gel bead in emulsions. Barcoded cDNA libraries were prepared following the manufacturer’s instructions using the Chromium Single Cell 3′ Reagent Kit version 3.1 (10X Genomics, PN-1000268). Amplified cDNA was quantified with the TapeStation D5000 (Agilent, 5067-5589). Gene expression libraries were constructed with 100 to 300 ng of amplified cDNA, and library size and concentration were assessed using the TapeStation D5000 kit. Sequencing was performed on the Illumina NovaSeq X Plus platform according to the manufacturer’s protocol.

### scRNA-seq data processing and filtering

Raw RNA-seq reads were aligned to human reference genome GRCh38 Gencode v47 gene annotation. Potential technical artifacts caused by ambient RNA contamination were corrected using DecontX from R package Celda (version 1.22.0) (*62*). Subsequently, low-quality cell barcodes were removed from the downstream analysis by filtering out cells with more than 15% mitochondrial gene expression, cells with less than 500 identified genes, those marked as doublets by the R package scDblFinder (version 1.20.2) (*63*), as well as cells with less than 1000 total counts. Overall, an average of 6232.33 cell barcodes per sample was included in the downstream analysis.

### Data normalization, integration, and clustering

In short, raw read counts were corrected for library size and log-normalized using NormalizeData function from Seurat. Next, to account for potential donor effects, we performed a principal components analysis of the top 4000 most variable genes, and Harmony (*64*) using the top 50 principal components was then applied to integrate cell-level expression. Top 30 Harmony components were used to compute the final integrated UMAP, and a neighborhood analysis and clustering of the integrated dataset was then performed using standard Seurat functions. To select an optimal clustering resolution, we used a clustering tree approach as implemented in R package Clustree version 0.5.1 (*65*).

### Cell type annotation and differential expression analysis

Cluster-specific marker genes were identified using the Wilcoxon rank-sum test. Next, false discovery rate (FDR) was estimated using Benjamini-Hochberg procedure (*66*). Genes with FDR < 0.05 were then used for cell type labeling. The annotation was then assigned using canonical markers from published endometrial atlases (*35*, *36*, *39*), examining their biological relevance with Human Protein Atlas (proteinatlas.org) (*67*) and GeneCards database (*68*).

Differentially expressed genes (DEGs) between hormone-treatment and nontreated cells were obtained using MAST (*69*) setting the sample donor, ratio of mitochondrial genes expressed per cell, number of counts and number of different genes expressed per cell as covariates. Overall, 16,273 genes were identified with FDR < 0.05 (data S1). Enrichment of gene ontology biological processes (*70*, *71*) within the identified DEGs was performed using WebGestaltR toolkit (version 0.4.6) (*72*).

### Comparison of ADOC gene expression with in vivo data

To compare the similarity between transcriptional profiles of the cells from the ADOC dataset and the in vivo data published in Marečková *et al.* (*35*), a similar score between identified cell types was calculated using the canonical correlation analysis method as implemented in Seurat (*73*). Similarity scores between ADOC and in vivo cell types are represented as a heatmap in Fig. 3G and fig. S3G.

### Frequency-based marker analysis

Cells were grouped by condition within each lineage, and gene expression was binarized at the single-cell level (count >0). For each marker gene, contingency tables were constructed, counting marker-positive and marker-negative cells per condition. Differences in detection frequency were assessed using two-sided Fisher’s exact tests. Odds ratios were calculated, and *P* values were adjusted for multiple testing using the Benjamini-Hochberg method.

### Analysis of cell-cell communication

To infer cell-cell communication among ADOC cell populations, we used CellChat (version 2.2.0) using CellChatDB as a receptor-ligand interaction database (*40*). Differential cell-cell communication between hormone-treated and untreated ADOC models was assessed using rankNet (alpha = 0.05).

### Software

All analyses were performed using R version v4.4.3. R packages were obtained with conda (https://docs.conda.io/en/latest/) using conda-forge (https://zenodo.org/records/4774217) and bioconda (*74*) channels, CRAN (https://www.r-project.org), and github.

### Microscopy and image analysis

The phase-contrast, fluorescent images, and time-lapse images were acquired using Leica Stellaris 5 and software associated (LASX version 4.8.0.28989). The 3D reconstructions and 2D planes were analyzed using FIJI 1.53k or Bitplane Imaris 9.7.0 software. For live imaging, embryos were scanned every hour at the upper, lower and middle plane of the embryo on the chip using a 10×/0.40 numerical aperture air objective.

Confocal immunofluorescence images of mouse and human blastocysts were acquired with a 10×/0.40 numerical aperture air-immersion objective or with a 25×/0.95 objective numerical water-immersion objective. For immunofluorescence images, optical sections from 0.57 to 1.5 μm were acquired.

In Fig. 2B, morphological changes of ESCs before and after decidualization were analyzed by changes in area and roundness. For this, images taken at random areas of the stromal compartment from three different chips were taken at 25×. For each cell, the area and roundness were measured manually outlining the cell boundary in ImageJ. Roundness was calculated by ImageJ as indicated in

$$\text{Roundness}=4\cdot \frac{\text{Area}}{\pi \cdot \left(\text{Major Axis}\right)2}$$

with values approaching 1 indicating more circular shapes.

In Fig. 2G, positive cells for PAEP and acetylated α-tubulin were identified on the basis of specific marker expression and divided by the total number of DAPI-stained nuclei within each field of view. Data are presented as the percentage of marker-positive cells relative to the total cell number.

Blastocyst adhesion time was determined by continuous monitoring of human embryos cultured on the microfluidic chip. Embryos were imaged at regular intervals to identify the time point at which adhesion onset occurred, defined as the moment of blastocoel collapse and stable contact with the epithelial layer. To validate adhesion, embryos were fixed in situ at different time points. Adhesion was confirmed when embryos remained immobile upon application of the fixative, indicating sufficient attachment to resist flow-induced displacement.

Blastocyst adhesion area in mouse and human was quantified by manually outlining the perimeter of each embryo in 2D slicers. The outer boundary of each embryo was manually delineated at the plane providing the best visibility of the contact region with the epithelial layer, and the corresponding surface area was calculated using the software’s measurement tools. For human blastocyst, “initial” indicated the area of the blastocyst once introduced in the chip. For mouse and human blastocyst, adhesion onset was defined morphologically during live imaging as the stage at which the blastocoel collapsed and optical density changed, indicating initial attachment. We referred to adhesion spreading when blastocyst expansion and lateral spreading across the epithelial surface of ADOC was observed.

To evaluate spatial positioning of embryo and epithelial cells, the tool surface was used to manually segment the nuclei in Imaris. Epithelial nuclei were segmented from DAPI cells lacking GATA3 expression, while embryonic nuclei were identified as GATA3/DAPI. The *Z* position (depth) of each segmented nucleus was extracted from the centroid coordinates provided by Imaris. These values were used to compare the vertical distribution of embryonic (GATA3) and epithelial nuclei and assess whether they occupied similar focal planes during embryo adhesion.

For human embryos, quantification of Δ*Z* distance to epithelium was measured by assessing the relative position of the embryo within the microfluidic device. For this, we generated 3D surfaces of the epithelial nuclei (DAPI) and the embryo (GATA3) and analyzed the Δ*Z* distance as the difference between the *Z* position (*Z* size of 0.57 μm) of the embryo and that of the average nuclei position in epithelial monolayer. Positive Δ*Z* values indicate that the embryo was located above the epithelial layer, while values close to zero indicate contact or integration with the epithelium.

### Statistical analysis

Statistical analyses were performed using Graphpad prism 8.1.1. Normality of the data distribution was assessed using the Shapiro-Wilk test. When assumptions of normality were met, comparisons between groups were performed using parametric tests [unpaired *t* test or one-way analysis of variance (ANOVA), as appropriate]. Otherwise, nonparametric tests were used (Mann-Whitney test). Reproducibility was confirmed by independent experiments.

For IGFBP-1 and PRL secretion and NTA analysis of EXOs and MVs, data were analyzed using a two-way ANOVA to assess the effects of cell type and time points, as well as treated and nontreated. When significant effects were detected, pairwise comparisons were performed using Bonferroni’s post hoc test to correct multiple comparisons.
