# Supplementary material for: Modeling human embryo adhesion using a microfluidic platform
Source: Sci Adv. 2026 Jul 10;12(28):eadz2249. doi: 10.1126/sciadv.adz2249 (PMC13353414; doi:10.1126/sciadv.adz2249)
Supplement: Supplementary file 1 — Figs. S1 to S6 Tables S1 to S4 Legends for movies S1 to S5 Legend for data S1 [file sciadv.adz2249_sm.pdf]

Science Advances
AAAS

Supplementary Materials for

Modeling human embryo adhesion using a microfluidic platform

Sofía Zaragozano *et al.*

Corresponding author: Carlos Simon, csimon@fundacioncarlossimon.com;
Felipe Vilella, fvilella@fundacioncarlossimon.com

Sci. Adv. **12**, eadz2249 (2026)
DOI: 10.1126/sciadv.adz2249

The PDF file includes:

Figs. S1 to S6
Tables S1 to S4
Legends for movies S1 to S5
Legend for data S1

Other Supplementary Material for this manuscript includes the following:

Movies S1 to S5
Data S1

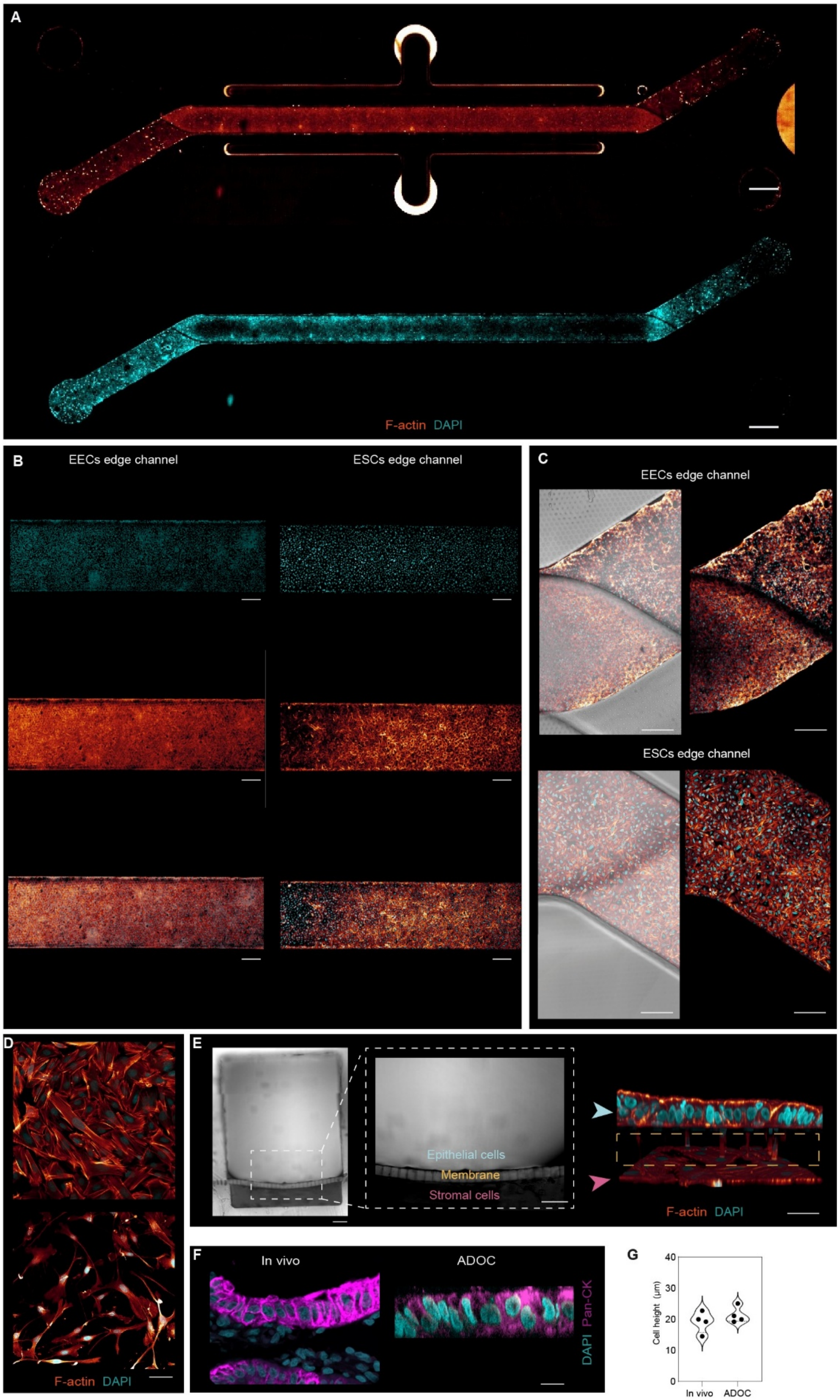
A
F-actin DAPI
B
EECs edge channel
ESCs edge channel
C
EECs edge channel
ESCs edge channel
D
F-actin DAPI
E
Epithelial cells
Membrane
Stromal cells
F-actin DAPI
F
In vivo
ADOC
DAPI Pan-CK
G
Cell height (µm)
40
30
20
10
0
In vivo
ADOC

Fig. S1. Structural and morphological characterization of ADOC. (A) Composite tile scan fluorescence image 8 days post-seeding, showing a fully confluent monolayer of organoid-derived endometrial epithelial cells and biopsy derived endometrial stromal cells. Scale bars, 1000 µm. Cells nuclei are shown in blue, cytoskeleton (F-Actin) is shown in orange. **(B and C)** Higher magnification views of the center and edge channels of the chip. Cells nuclei are shown in blue, cytoskeleton (F-Actin) is shown in orange. Scale bars, 300 µm **(B)**, 200µm **(C)**. (**D**) Representative images showing variability in stromal cells morphology between donors with DAPI (blue) and F-actin (red) staining. Scale bar 50 µm**. (E)** Representative chip cross-section illustrating the spatial organization of the epithelial and stromal compartments separated by a porous membrane. Brightfield tile scan (right) and corresponding zoom (middle) show the cross-sectional architecture. A 3D rendering (left) reveals a polarized epithelial layer on the upper side of the membrane and stromal cells on the opposite side within the lower channel. The membrane defines the physical separation between compartments (yellow dashed square). Apparent vertical "bridges" correspond to optical projections of membrane pores and aligned stromal cell extensions. Scale bar, right and middle panel 100 µm, left panel 50 µm. **(F)** Representative images of epithelial organization in vivo (left panel) and in ADOC (Right panel) stained for Pan-Cytokeratin (Pan-CK, purple) and DAPI (cyan). Scale bars: 10 and 15 µm, respectively. (**G)** Violin plot quantification of epithelial cell height in vivo human endometrium and ADOC. Data represents the average cell data of 4 biological replicates and 4 independent chips from 3 donors (ns, p>0.05, unpaired t-test).

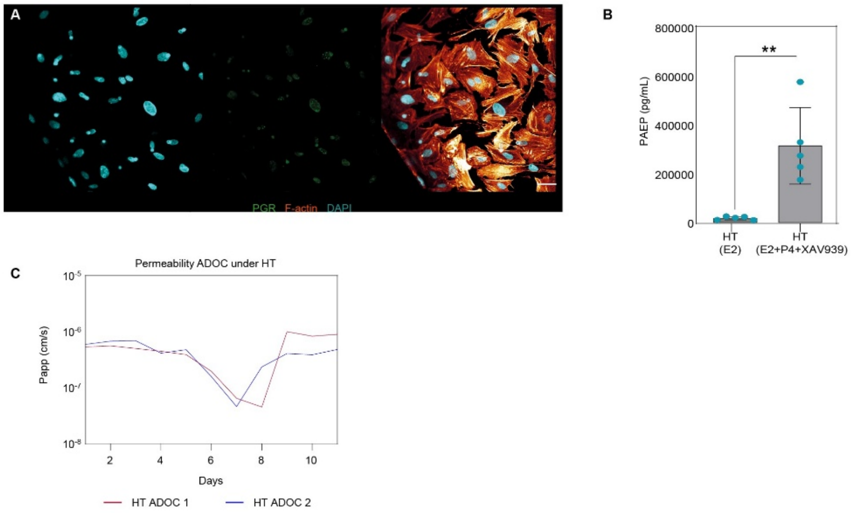

Fig. S2. Hormonal response in ADOC. (A) Representative immunofluorescence images of stromal cells demonstrating increased expression of progesterone receptors (PGR, green) in response to hormonal treatment, along with DAPI (blue) and F-actin (red) staining. Scale bars, 40 µm. **(B)** PAEP secretion of epithelial cells is presented as the mean ± S.D., of 5 chips from 3 independent donors. **$p<0.01$ by unpaired t-test. **(C)** Changes in apparent permeability between the epithelium compartment of two different chips (n=2).

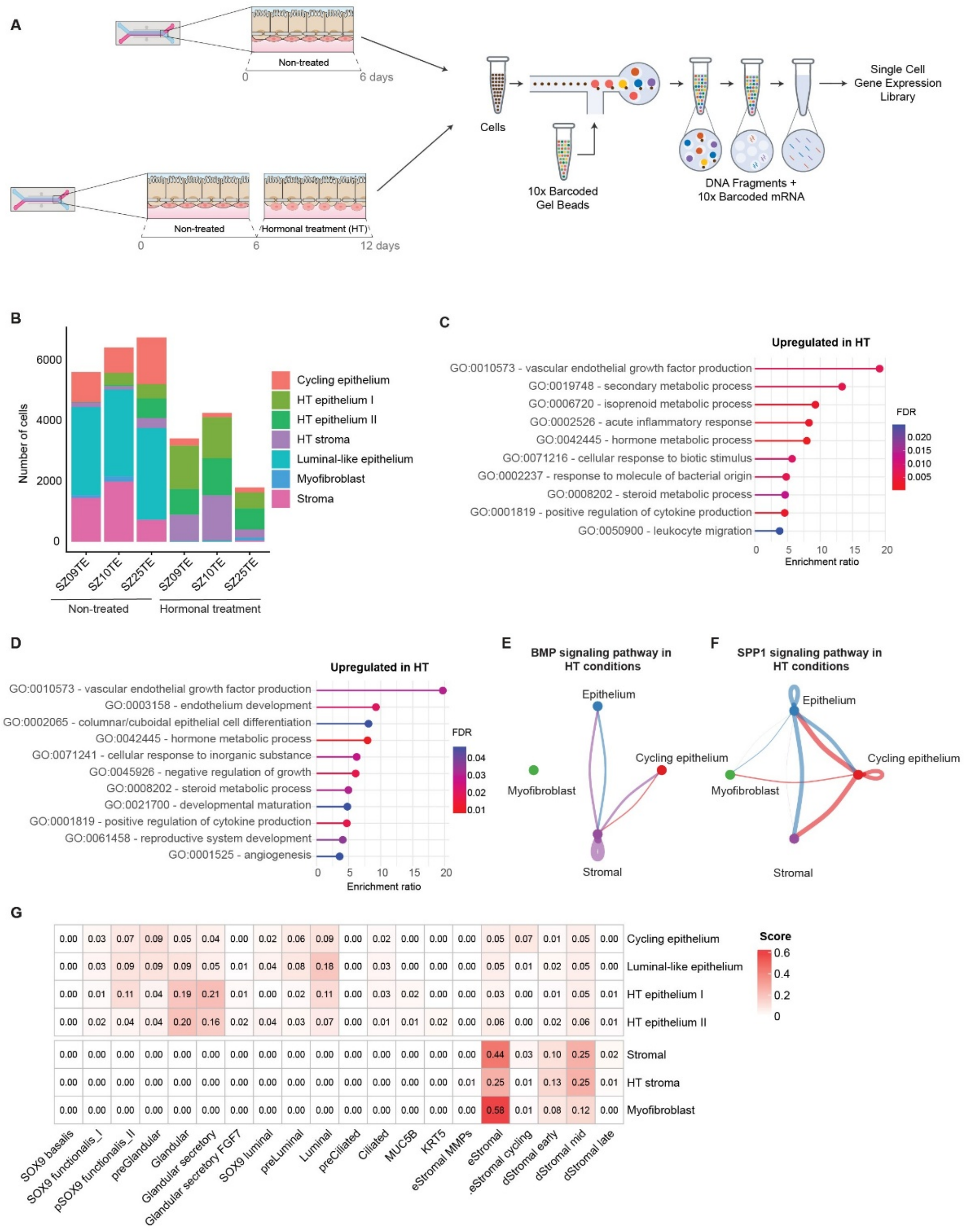

Fig. S3. Single-cell transcriptomics profiling of ADOC. **(A)** Schematic representation of the scRNA-seq procedure in ADOC. **(B)** Bar plot showing the numbers of cells sequenced per sample and their distribution across the identified cell populations. We performed scRNA-seq in 6 independent chips from 3 donors. **(C)** Over-representation analysis of GO terms enriched in differentially expressed genes from the HT (I + II) epithelium compared to the luminal-like epithelium. **(D)** Over-representation analysis of GO terms enriched in differentially expressed genes from the HT stroma compared to the stromal population. **(E)** Chord plot displaying cell–cell communication networks showing active BMP pathway under HT conditions. Edge thickness

corresponds to communication strength. **(F)** Chord plot displaying cell–cell communication networks showing active SPP1 pathway under HT conditions. Edge thickness corresponds to communication strength. **(G)** Cell-type correspondence between in vitro and in vivo endometrial datasets. Heatmap showing the average prediction scores of in vitro cell types mapped to in vivo reference cell types (35). Rows represent in vitro cell types, and columns correspond to in vivo identities. The color scale indicates the mean prediction score. All epithelial and stromal cells (35) are shown independently of their prediction score.

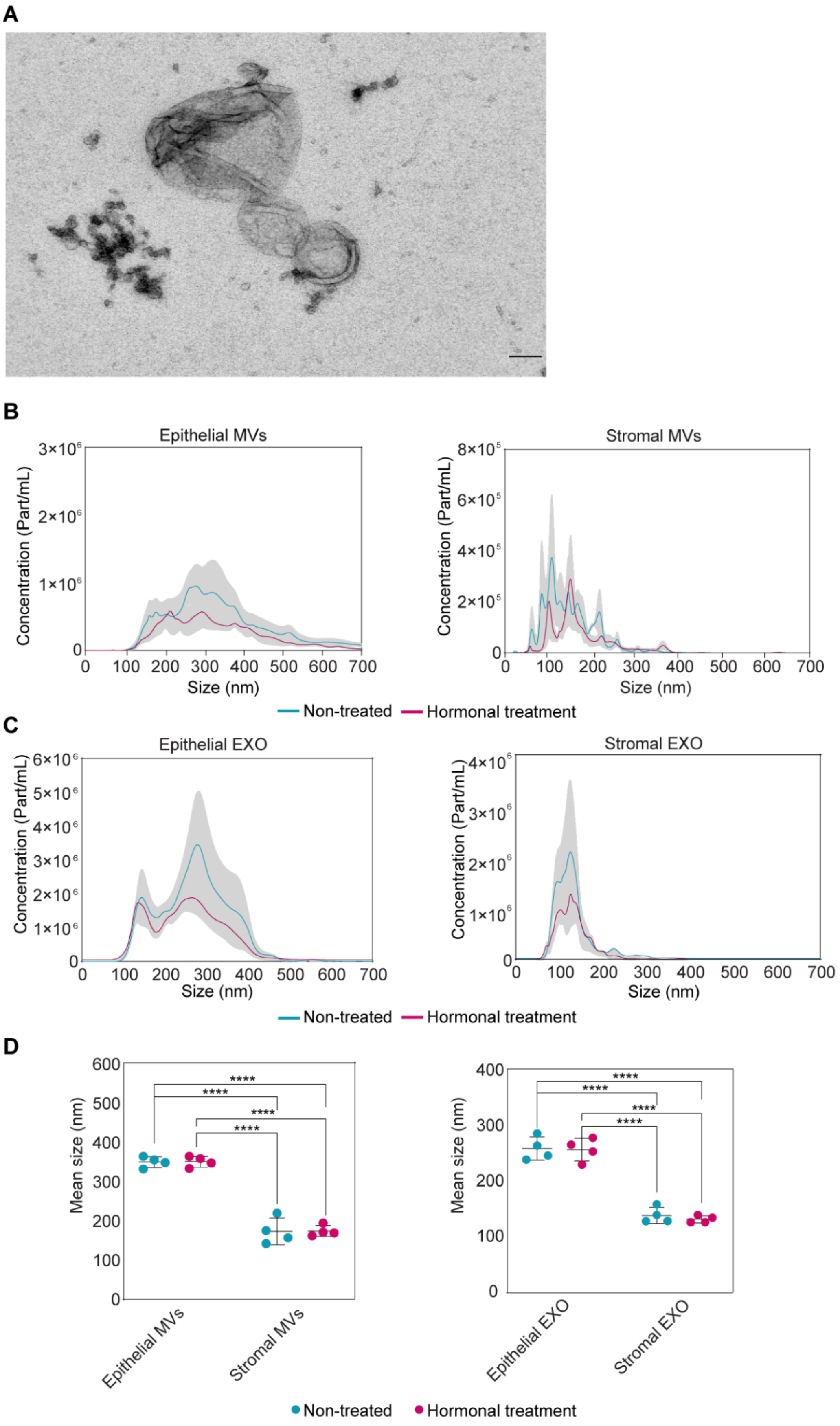
A
B
Epithelial MVs
Stromal MVs
Concentration (Part/mL)
Size (nm)
Non-treated
Hormonal treatment
C
Epithelial EXO
Stromal EXO
Concentration (Part/mL)
Size (nm)
Non-treated
Hormonal treatment
D
Mean size (nm)
****
Epithelial MVs
Stromal MVs
Epithelial EXO
Stromal EXO
Non-treated
Hormonal treatment

Fig. S4. Morphology and nanoparticle tracking analysis of isolated EVs. (A) Representative transmission electron microscopy (TEM) images showing apoptotic bodies (ABs) present in the medium of the chip. Scale bar, 100 nm. **(B)** Size distribution of microvesicles (MVs) as a function of particle concentration. **(C)** Size distribution of exosomes (EXOs) as a function of particle concentration. **(D)** Comparison of mean particle sizes for MVs and EXOs in media from hormone-treated and non-treated chips.

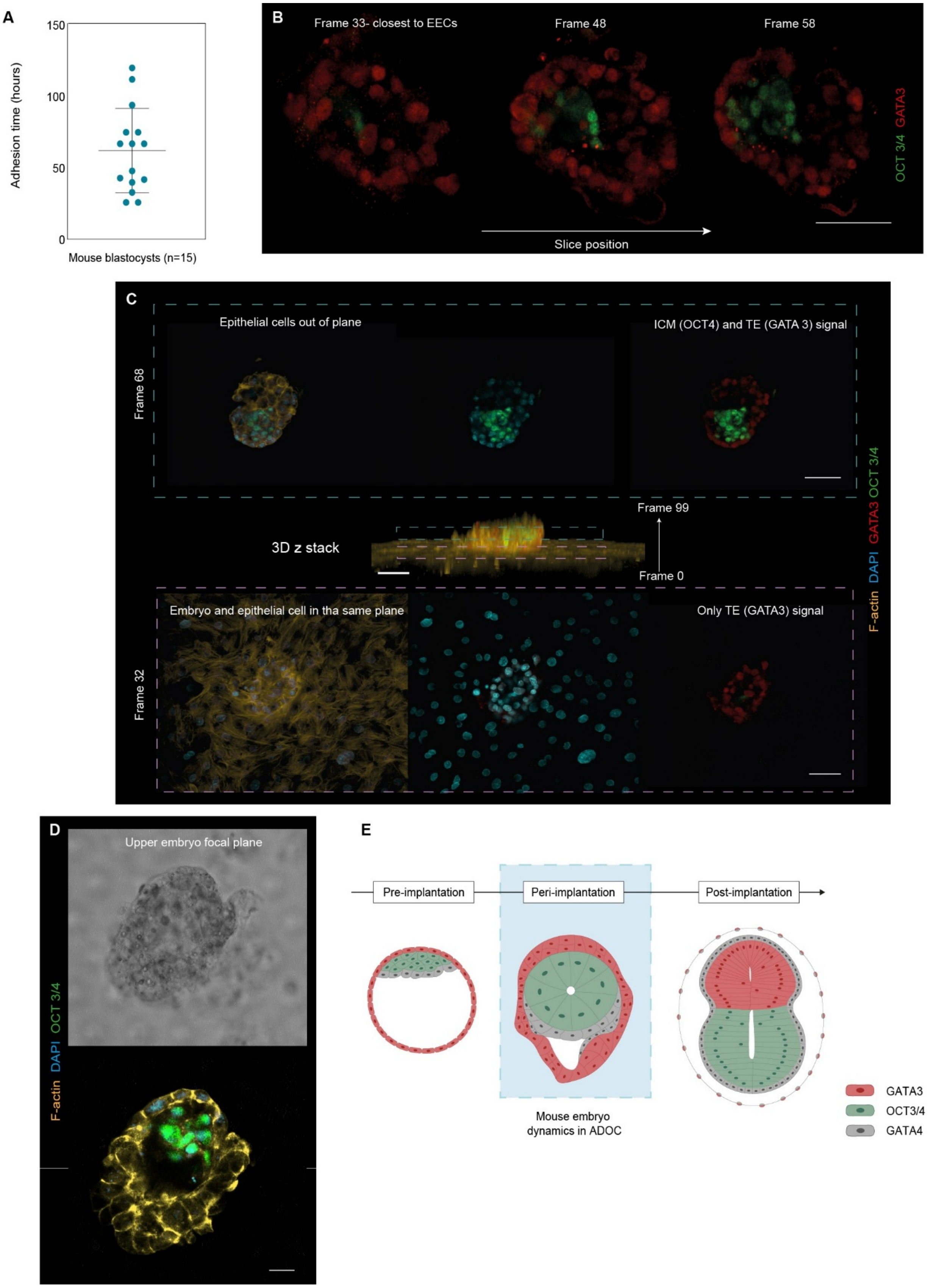
A
Adhesion time (hours)
150
100
50
0
Mouse blastocysts (n=15)
B
Frame 33- closest to EECs
Frame 48
Frame 58
OCT 3/4 GATA3
Slice position
C
Epithelial cells out of plane
ICM (OCT4) and TE (GATA 3) signal
Frame 68
3D z stack
Frame 99
Frame 0
Embryo and epithelial cell in tha same plane
Only TE (GATA3) signal
Frame 32
F-actin DAPI GATA3 OCT 3/4
D
Upper embryo focal plane
F-actin DAPI OCT 3/4
E
Pre-implantation
Peri-implantation
Post-implantation
Mouse embryo
dynamics in ADOC
GATA3
OCT3/4
GATA4

Fig. S5. Adhesion timing, spatial orientation and developmental dynamics of mouse blastocysts in ADOC. (A) Early adhesion time distribution of 15 embryos introduced into the chip, showing variability in the onset of adhesion. Data is presented as the mean±S.D. (n=15). **(B)** Z-stack slices showing adhesion initiation by the trophectoderm. Left frame: plane closest to the epithelial layer (EECs). Right frame: plane farthest from the epithelial layer. Staining: ICM (OCT3/4, green) and trophectoderm (GATA3, red). Scale bar, 70µm. **(C)** Representative imaging of the same mouse embryo shown in Fig. 5D, illustrating how embryo–epithelium interactions and marker localization depend on the focal plane. A 3D Z-stack reconstruction (~100 optical sections) is shown (middle panel). The lower panel (frame 32, aligned with the epithelial layer) shows epithelial cells surrounding the embryo (F-actin, yellow), with enrichment of $GATA3^{+}$ trophectoderm cells (red) at the contact interface. The upper panel (frame 68) shows the inner cell mass ($OCT4^{+}$, green), while the epithelial layer is out of focus and not visible. Scale bars, 50 µm. **(D)** Representative brightfield and immunofluorescence images of the embryo shown in Fig. 5H. Cavity remodeling is observed at the apical region of the embryo, while the porous membrane and epithelial layer are out of focus. F-actin (yellow), DAPI (blue), and OCT3/4 (green). Scale bar, 20µm**.** **(E)**Schematic representation of mouse embryo developmental stages illustrating trophectoderm (GATA3), inner cell mass (OCT3/4), and primitive endoderm (GATA4) organization capture in ADOC.

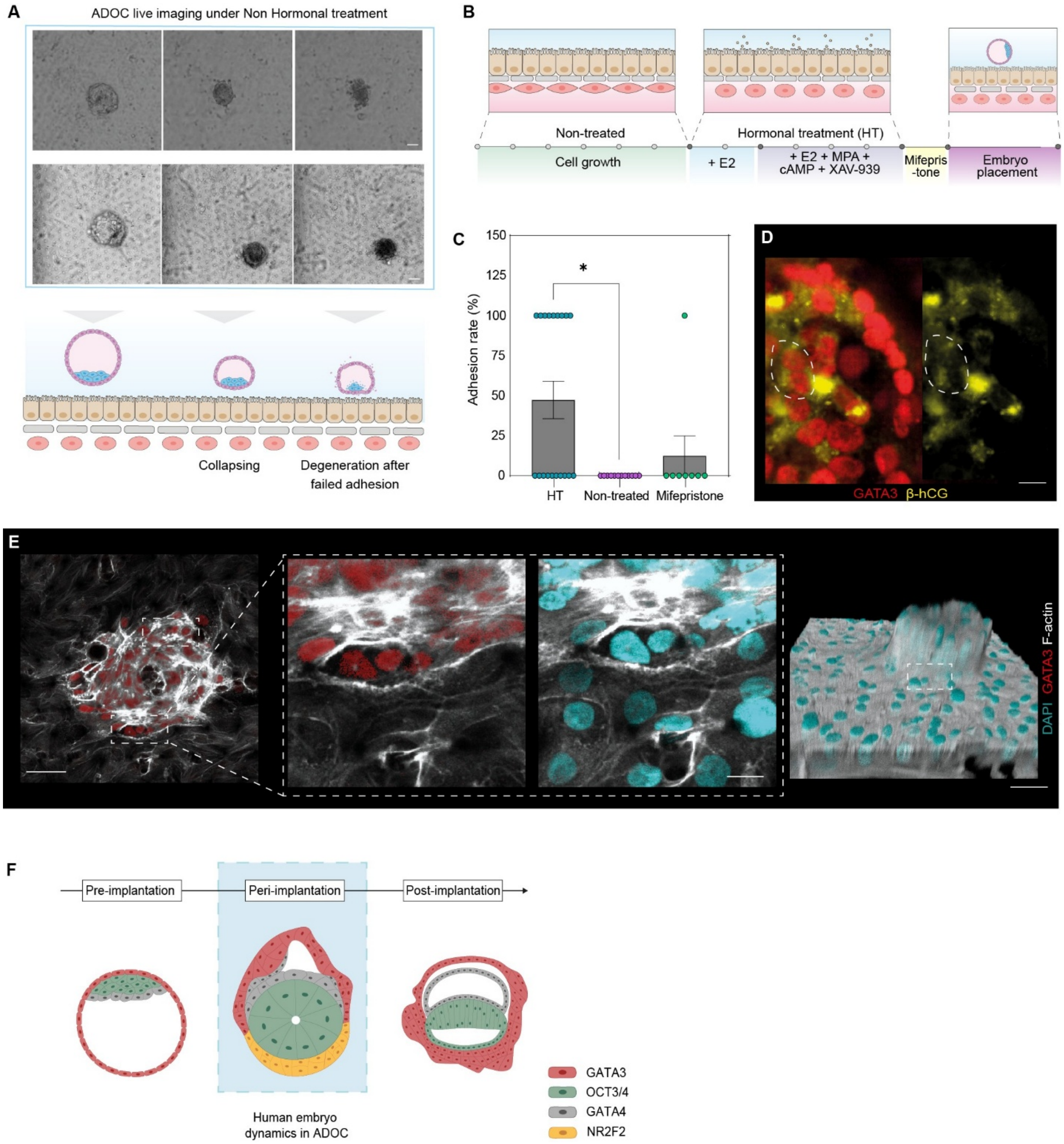

Fig. S6. Human embryo adhesion dynamics under hormone-free, contraceptive and hormone -stimulated conditions. (A) Representative time-lapse images of human blastocysts cultured in ADOC under hormone-free conditions. Embryos exhibited cycles of contact, collapse, and eventual degeneration following failed adhesion. The schematic below illustrates the sequence of events observed. **(B)** Experimental scheme of the contraceptive assay. After 6 days of cell growth, cultures were treated with E2, cAMP, MPA, and XAV939 to induce receptivity. Mifepristone was subsequently added for 24 hours prior to embryo placement to assess its effect on implantation. **(C)** Embryo adhesion rate (%) under hormonal treatment (HT, n=19), non-treated

(n=12), and mifepristone-treated conditions (n=8). Data are shown as mean ± SEM. Each dot represents one single embryo adhesion experiment. Statistical analysis was performed using one-way ANOVA Kruskal–Wallis test; $p < 0.05$. **(D)** High-magnification images showing co-localization of β-hCG (yellow) with GATA3+ trophoblast cells (red) (dashed region), Scale bar 15μm. **(E)** Representative confocal images of an adhered human embryo showing GATA3+ trophoblast cells (red) and F-actin (white), with nuclei stained by DAPI (cyan) (Right panel). High-magnification views (dashed boxes) and orthogonal 3D reconstruction (left panel) demonstrate the presence of multinucleated GATA3+ cell structures localized within the same epithelial plane, supporting the formation of multinucleated trophoblast domains. Scale bars 50μm (Right and left panels), 15μm (middle, dashed panels). **(F)**Comparative schematic of pre-, peri-, and post-human embryo development, describing in detail the stage of human embryo dynamics captured in ADOC (blue square, NR2F2 is only highlighted at the ADOC stage).

Table S1. Endometrial epithelial organoid (EEO) medium composition

**Product**	**Concentration**	**Supplier**	**Catalog #**
DMEM/F12		Gibco	11330032
R-Sponding	200 ng/mL	R&D systems	120-38
Noggin	100 ng/mL	R&D systems	6057-NG-100
B-27 Supplement	2%	Fisher scientific	12587010
N2 Supplement	1%	Fisher scientific	17502048
Insulin transferrin selenium	1%	Life Technologies	41400045
Penicilin /Streptomycin	1%	Fisher scientific	15140122
Nicotinamide	5 mM	Merck	72340-100G
A83-01	0.5 µM	Merck	SML0788-5MG
N-Acetyl L-cysteine	1.25 mM	Merck	A7250-50G
Epidermal growth factor (EGF)	50 ng/mL	R&D systems	236-EG-01M
Basic Fibroblast Growth Factor (bFGF)	2 ng/mL	Fisher scientific	PHG0264
FGF-10	50 ng/mL	Prepotech	100-26
p38 inhibitor	10 µM	Merck	S7067
Y-27632	10 µM	Merck	SCM075

Table S2. Embryo medium composition

Product	Concentration	Supplier	Catalog #
DMEM/F12		Gibco	21041025
FBSi	10%	Biowest	S181B
B-27 Supplement	2X	Fisher scientific	12587010
N2 Supplement	1X	Fisher scientific	17502048
Insulin transferrin selenium	1X	Life Technologies	41400045
Penicilin /Streptomycin	1%	Fisher scientific	15140122
N-Acetyl L-cysteine	1.25 mM	Merck	A7250-50G
Essential Amino Acids	1X	Gibco	11130051
No essential Amino Acids	1X	Gibco	11140050
17β-estradiol (E2)	10 mM	Merck	E2758-250MG
Cyclic adenosine monophosphate (cAMP)	0.5 mM	Merck	B7880-10MG
Medroxyprogesterone acetate (MPA)	1 µM	Merck	M1629

From Rawlings, et al. *eLife* **10**, e69603 (2021). Reference (*15*) in main manuscript.

Table S3. Primary antibodies

**Antibody**	**Supplier**	**Catalog #**
ZO-1	Invitrogen	33-9100
Vimentin	Biotechne	AF2105
Acetylated α-tubulin	Merck	T7451-25UL
PAEP	Abcam	ab270525
Pan-Cytokeratin	Abcam	ab217916
IGFBP-1	Invitrogen	PA5-61-388
Prolactin	OriGene	CF500719
GATA3	Invitrogen	14-9966-82
GATA4	Invitrogen	14-9980-2
OCT3/4	Santa Cruz	SC5279
GATA 3	R&D systems	AF2605
PGR	Cell signalling technology	D8Q2J
hCG	Abcam	ab9582
NR2F2	Abcam	ab211776
Pan-Cytokeratin	Abcam	ab217916

Table S4. Secondary antibodies

Antibody	Supplier	Catalog #
Anti-mouse 488	Invitrogen	A21202
Anti-rabbit 555	Invitrogen	A31572
Anti-goat 647	Invitrogen	AF1924
Anti-rat 488	Invitrogen	A21208
Anti-rat 647	Invitrogen	A48272
Anti-mouse 647	Invitrogen	A32787
Anti-mouse 568	Invitrogen	A10037

Movie S1. Mouse blastocyst adhesion in ADOC platform over time.
Brightfield imaging showing media flushing across the chip channel, confirming embryo adhesion. Scale bar, 50 µm.

Movie S2. Inner cell mas (ICM) and trophectoderm (TE) spatial organization in mouse blastocyst.
3D confocal slicer showing mouse blastocyst adhered to the epithelial layer. GATA3+ (red) TE cells make initial contact and spread over the epithelial monolayer, while OCT4+ (green) ICM cells remain apically located.

Movie S3. Time-lapse brightfield imaging of human blastocyst adhering onto ADOC.
Scale bar, 100 µm.

Movie S4. Time-lapse brightfield imaging of human blastocyst into ADOC in non-treated conditions. Scale bar 50 µm.

Movie S5. 3D reconstruction of human embryo embedded in the ADOC epithelial channel.
GATA3 trophectoderm (red), OCT4 inner cell mass (green), and DAPI (blue). Scale bar, 50 µm.

Data S1 (separate file)
snRNA-seq information. DEGs per cluster; ORA Epithelial HT vs Epithelial No HT; ORA Stromal HT vs Stromal No HT; DE Epithelial HT vs DE Epithelial No-HT; DE stroma HT vs DE stroma No-HT; In vivo integration_HECA, Cell counts by patients and cell type; Cell-Cell communication No Ht ; Cell-Cell communication HT
